# Impact of must clarification treatments on chemical and sensory profiles of kiwifruit wine

**DOI:** 10.1038/s41538-024-00280-z

**Published:** 2024-06-25

**Authors:** Di Huang, Wenjing Fan, Ruisen Dai, Yao Lu, Yanlin Liu, Yuyang Song, Yi Qin, Ying Su

**Affiliations:** 1 College of Enology, Northwest A&F University, Yangling, China; 2 Engineering Research Center for Viti-Viniculture, National Forestry and Grassland Administration, Yangling, China

**Keywords:** Technology, Engineering, Engineering, Chemistry

## Abstract

This study examined the effect of various clarification treatments on the physicochemical properties, volatile compounds, and sensory attributes of kiwi wines produced from five different kiwifruit (*Actinidia deliciosa*) varieties. The degree of clarification had a minimal impact on physicochemical parameters, including the content of residual sugar, ethanol, volatile acid, titratable acidity (except for the kiwifruit variety ‘Qinmei’), and the pH value. However, wines made from unclarified juices (muddy juice and pulp) displayed a higher glycerol content than those made from clarified juices. The cluster heat map and principal component analyses (PCA) demonstrated that kiwi wines produced from clarified kiwi juices possessed a higher ester content, whereas muddy juice and pulp wines contained elevated levels of higher alcohols. Quantitative descriptive analysis (QDA) indicated that clarified juice wines outperformed muddy juice and pulp wines in terms of purity, typicality, harmony, intensity, and freshness, with negligible differences in terms of palate acidity. Moreover, the clarified juice wines featured more characteristic kiwi wine aromas (kiwifruit, passionfruit, and pineapple) compared with that of the muddy juice and pulp wines, which exhibited an increased grassy flavour. Although the 100-NTU kiwifruit juice-fermented wine did not show an advantage in the cluster heat map and PCA, it presented better freshness, typicality, and intensity in the QDA, as well as a more passionfruit aroma. Based on the orthogonal partial least-squares discriminant analysis, *A. deliciosa* ‘Xuxiang’ was deemed to be the most suitable variety for vinification. This study provides crucial insights for enhancing the production of high-quality kiwi wine.

## Introduction

Kiwifruit is a rich source of vitamins, flavonoids, carotenoids, and minerals essential for the human body^1^. Nutrient-rich kiwi wine not only expands the kiwi wine value chain but is also anticipated to be appreciated by consumers as a novel fruit wine^2^.

The sensory quality of kiwi wine is a significant aspect of its overall quality and profoundly influences consumer preference^3^. However, marketed kiwi wines widely suffer from flat taste characteristics and lack of aroma profiles, which have hindered the growth of the sector. Kiwi wines have not been extensively researched and most of the current research focuses on the impacts of the yeast strain, fermentation temperature, and kiwi fruit variety^2,4,5^, with relatively less attention paid to the development of the actual brewing technology. Non-*Saccharomyces cerevisiae* fermentation has received considerable attention in improving the aroma quality of fruit wines^6^; however, most of them are poorly suited for large-scale commercial applications owing to their difficulty in fermentation alone or their production of various defective aroma profiles^7^. In contrast, the fermentation technology choice provides better broad applicability.

Some solid particles in the juice can significantly influence the aroma composition and organoleptic properties of fruit wines^8,9^. These substances can be effectively removed by clarification of the freshly pressed juice^10^, which is a crucial aspect of the brewing technology for white wine. The colour of fruit wines is quantified in the equidistant L*a*b* colour space, however, clarity is often denoted by their turbidity values, expressed in nephelometric turbidity units (NTU). The turbidity in turn affects the volatile compound content. Wines fermented using low turbidity must tend to exhibit elevated levels of C6, C7, and C8 fatty acids, which have the potential to enhance the fermentation aromas of the wine^10^. In contrast, fermentation involving high turbidity must result in wines containing reduced ethyl ester levels and increased levels of isobutanol and isoamyl alcohol^11,12^. At low thresholds, esters contribute to pleasant fruity and floral aromas, which significantly influence the intensity and persistence of the wine’s aroma^13^. The effect of elevated levels of higher alcohols on wine aroma is similar to that of fatty acids in which both exhibit a dose-dependent behaviour^14^. Excessive amounts of higher alcohols such as isobutanol, isoamyl alcohol and phenylethanol impart more ‘chemical’ and ‘fatty’ odours to the wine. As the concentration of fatty acids increases, the wine’s aroma changes from ‘fruity’ and ‘cheesy’ to ‘fatty liver’ and even ‘rancid’^13^. Notably, in our previous study^15^, kiwifruit wines were found to be more prone to exceeding their organoleptic thresholds for higher alcohols, which impaired their sensory aroma. In contrast, fatty acid concentrations exceeding the thresholds leading to the production of defective aromas were not reported.

Turbidity also influences the release of macromolecules from the *S. cerevisiae*; juice clarification inevitably eliminates important nutrient sources (unsaturated and saturated fatty acids, nitrogenous compounds, and polysaccharides) that are used by the yeast during the alcoholic fermentation (AF) process^10^. In low-turbidity juices, yeast tends to yield more extracellular and cell wall polysaccharide compounds during AF^8^, which directly affect the taste and sweetness of the wine, albeit making a limited contribution to the nutritional value^9^. Notably, excessive clarification during white grape wine fermentation is considered a significant factor contributing to sluggish fermentation^16,17^, which should be avoided. Clarified must-yield wines with reduced browning rates^18^, whereas highly turbid fermented mash tends to yield wines with pronounced reductive odours^19^. However, in apple juice processing, turbid juices possess significantly higher antioxidant capacities than clarified juices^20^. These findings suggest that minimising additional manipulation with less manual intervention during wine-making could help to prevent oxidation during turbid juice fermentation, thus meeting consumer expectations for a product with high antioxidant properties^21^.

The impact of juice clarification on the organoleptic quality of kiwi wine remains unexplored, having only been examined in a limited manner in grape wine^19^. To fill this gap, we investigated the influence of various clarification levels applied to kiwifruit juice on the quality of the kiwi wine produced by analysing the physicochemical parameters, volatile components, and sensory qualities of five kiwifruit varieties from Shaanxi, China. The findings of this investigation will aid in evaluating the quality of kiwi wine under varying clarification conditions for the fermentation mash and offer theoretical insights into improving kiwi wine production.

## Results and discussion

### Effects of varying clarification levels on the physicochemical properties of kiwi wine

All the samples in this study were able to start and complete fermentation successfully (Supplementary Fig. 1), with the “Qinmei” variety having the longest fermentation duration and the “Huayou” variety having the shortest, however, there was no significant difference between the residual sugar contents at the end of fermentation. In addition, no significant differences were observed in alcohol, volatile acid, and pH across different degrees of clarification within all five varieties (Table 1), which is consistent with the findings by Cheng and Watrelot^22^ in studying the effects of different fining agents on wines. This suggests that the growth activity of *S. cerevisiae* CEC01 used in this study was unaffected by the varying clarification levels.

**Table 1:** Physicochemical properties of all kiwi wines

Variety	Sample	Reducing sugar, (g/L)	Alcohol content, (%vol)	Titratable acidity, (g/L)	Volatile acids, (g/L)	pH	Glycerol, (mg/L)	Ammonium, (mg/L)	CIELab		
									L*	a*	b*
Xuxiang	100 NTU	3.61 ± 0.11^a^	11.77 ± 0.01^a^	12.60 ± 0.30^c^	0.14 ± 0.02^a^	3.52 ± 0.03^a^	5.10 ± 0.28^c^	4.40 ± 1.13^a,b^	99.07 ± 0.04^a^	−0.44 ± 0.00^d^	3.16 ± 0.01^d^
	200 NTU	3.47 ± 0.08^a^	10.93 ± 0.02^a^	11.83 ± 0.25^b^	0.14 ± 0.02^a^	3.37 ± 0.17^a,b^	5.60 ± 0.00^b^	5.40 ± 2.55^a,b^	98.3 ± 0.01^c^	−0.35 ± 0.00^c^	3.74 ± 0.01^c^
	300 NTU	3.64 ± 0.22^a^	11.50 ± 0.02^a^	12.67 ± 7.70^b,c^	0.14 ± 0.02^a^	3.29 ± 0.04^b^	5.95 ± 0.07^b^	7.05 ± 1.06^a^	97.91 ± 0.01^d^	−0.30 ± 0.00^b^	3.93 ± 0.01^bc^
	Muddy juice	3.66 ± 0.05^a^	11.84 ± 0.01^a^	13.27 ± 0.25^b, c^	0.20 ± 0.04^a^	3.22 ± 0.04^b^	7.35 ± 0.07^a^	3.40 ± 0.42^c^	98.39 ± 0.01^b^	−0.36 ± 0.01^c^	4.03 ± 0.01^b^
	Pulp	3.41 ± 0.04^a^	11.55 ± 0.01^a^	13.12 ± 0.25^a^	0.17 ± 0.02^a^	3.22 ± 0.05^b^	7.25 ± 0.07^a^	3.90 ± 0.28^a,b^	97.04 ± 0.06^e^	−0.20 ± 0.04^a^	5.03 ± 0.24^a^
Huayou	100 NTU	3.86 ± 0.10^b^	10.60 ± 0.14^b^	13.31 ± 1.81^a^	0.24 ± 0.01^a^	3.61 ± 0.01^a,b^	4.7 ± 0.14^a^	5.00 ± 1.70^b^	98.14 ± 0.00^a^	−1.78 ± 0.01^c^	8.29 ± 0.05^e^
	200 NTU	3.96 ± 0.07^b^	11.25 ± 0.07^a^	12.61 ± 1.42^a^	0.20 ± 0.01^a,b^	3.63 ± 0.01^a^	5.05 ± 0.21^a^	6.55 ± 0.78^b^	97.68 ± 0.02^b^	1.755 ± 0.01^c^	8.84 ± 0.06^d^
	300 NTU	3.95 ± 0.14^b^	10.95 ± 0.07^b^	13.50 ± 1.23^a^	0.21 ± 0.02^a,b^	3.52 ± 0.05^c^	5.3 ± 0.57^a^	7.50 ± 0.42^b^	97.70 ± 0.01^b^	−1.78 ± 0.03^c^	9.05 ± 0.04^c^
	Muddy juice	3.91 ± 0.09^b^	11.30 ± 0.14^a^	14.21 ± 0.06^a^	0.20 ± 0.00^b^	3.48 ± 0.01^c^	5.4 ± 0.14^a^	13.40 ± 3.25^a^	97.10 ± 0.04^c^	−1.695 ± 0.01^b^	9.27 ± 0.08^b^
	Pulp	4.22 ± 0.02^a^	11.05 ± 0.21^a^	12.83 ± 1.06^a^	0.22 ± 0.02^a,b^	3.48 ± 0.01^c^	5.3 ± 0.00^a^	16.50 ± 1.13^a^	94.74 ± 0.04^d^	1.295 ± 0.01^a^	11.11 ± 0.01^a^
Hayward	100 NTU	3.73 ± 0.06^a^	11.36 ± 0.00^b^	15.29 ± 0.08^a^	0.16 ± 0.08^a^	3.25 ± 0.01^a^	5.7 ± 0.10^c^	5.45 ± 0.25^a^	99.14 ± 0.00^a^	−0.525 ± 0.01^d^	3.41 ± 0.01^d^
	200 NTU	3.48 ± 0.42^a^	11.65 ± 0.07^a^	15.18 ± 0.05^a^	0.15 ± 0.08^a^	3.23 ± 0.01^a,b^	6.05 ± 0.15^c^	7.00 ± 2.10^a^	98.96 ± 0.00^a^	−0.475 ± 0.01^c^	3.45 ± 0.01^d^
	300 NTU	2.69 ± 0.24^b^	11.17 ± 0.02^c^	15.34 ± 0.13^a^	0.21 ± 0.04^a^	3.25 ± 0.01^a^	6.25 ± 0.15^c^	6.05 ± 0.45^a^	98.85 ± 0.02^b^	−0.465 ± 0.03^b^	3.60 ± 0.02^b^
	Muddy juice	3.47 ± 0.39^a^	11.74 ± 0.01^a^	15.20 ± 0.04^a^	0.15 ± 0.00^a^	3.21 ± 0.01^b^	7.60 ± 0.30^b^	5.10 ± 1.70^a^	96.72 ± 0.00^a^	−0.43 ± 0.01^bc^	5.54 ± 0.01^c^
	Pulp	3.18 ± 0.12^a,b^	11.66 ± 0.01^a^	15.08 ± 0.23^a^	0.15 ± 0.04^a^	3.22 ± 0.01^b^	8.60 ± 0.20^a^	6.85 ± 0.75^a^	93.14 ± 0.33^c^	−0.135 ± 0.01^a^	7.95 ± 0.11^a^
Qinmei	100 NTU	4.73 ± 0.10^a^	10.90 ± 0.14^a^	16.38 ± 0.33^a^	0.11 ± 0.01^a^	3.13 ± 0.01^a,b^	4.20 ± 0.30^c^	4.70 ± 0.20^a^	99.51 ± 0.01^c^	−0.60 ± 0.00^c^	2.99 ± 0.00^d^
	200 NTU	4.21 ± 0.30^a,b^	11.25 ± 0.07^a^	16.46 ± 0.33^a^	0.15 ± 0.07^a^	3.11 ± 0.07^b^	4.60 ± 0.10^b,c^	5.50 ± 0.20^a^	99.65 ± 0.01^a^	−0.56 ± 0.01^c^	2.96 ± 0.00^e^
	300 NTU	3.86 ± 0.08^b^	11.23 ± 0.04^a^	16.15 ± 0.06^a^	0.13 ± 0.04^a^	3.12 ± 0.04^b^	4.95 ± 0.05^b^	5.20 ± 0.80^a^	99.62 ± 0.00^b^	−0.63 ± 0.00^d^	3.09 ± 0.00^c^
	Muddy juice	3.92 ± 0.14^b^	11.10 ± 0.14^a^	13.09 ± 0.77^b^	0.19 ± 0.02^a^	3.19 ± 0.02^a^	6.75 ± 0.05^a^	5.40 ± 0.50^a^	98.72 ± 0.01^d^	−0.45 ± 0.00^b^	3.47 ± 0.01^b^
	Pulp	3.66 ± 0.08^b^	11.10 ± 0.28^a^	13.8 ± 0.56^b^	0.19 ± 0.02^a^	3.19 ± 0.02^a^	6.45 ± 0.15^a^	8.40 ± 2.30^a^	98.41 ± 0.00^e^	−0.33 ± 0.01^a^	3.54 ± 0.01^a^
Yate	100 NTU	3.96 ± 0.28^b^	11.35 ± 0.07^a^	12.53 ± 0.11^a^	0.22 ± 0.02^a^	3.44 ± 0.01^c^	6.00 ± 0.30^b^	9.05 ± 0.45^a^	97.55 ± 0.09^b^	−0.87 ± 0.13^c^	4.64 ± 0.16^c^
	200 NTU	4.54 ± 0.06^a,b^	11.35 ± 0.07^a^	11.26 ± 0.71^b^	0.17 ± 0.01^b^	3.52 ± 0.01^a^	6.65 ± 0.05^a^	8.90 ± 0.30^a^	98.17 ± 0.01^a^	−0.81 ± 0.00^c^	4.83 ± 0.04^c^
	300 NTU	5.01 ± 0.46^a^	11.65 ± 0.07^a^	12.04 ± 0.71^a,b^	0.18 ± 0.01^b^	3.46 ± 0.01^b,c^	6.10 ± 0.10^b^	8.95 ± 0.45^a^	98.01 ± 0.03^a^	−0.78 ± 0.02^c^	4.69 ± 0.05^c^
	Muddy juice	4.46 ± 0.34^a,b^	11.65 ± 0.35^a^	12.76 ± 0.03^a^	0.17 ± 0.01^b^	3.47 ± 0.00^b^	6.80 ± 0.00^a^	7.10 ± 0.30^a^	96.37 ± 0.02^c^	−0.55 ± 0.00^b^	5.76 ± 0.01^b^
	Pulp	4.08 ± 0.28^b^	11.75 ± 0.07^a^	13.09 ± 0.64^a^	0.17 ± 0.00^b^	3.50 ± 0.00^a^	6.90 ± 0.10^a^	8.30 ± 1.20^a^	90.21 ± 0.16^d^	0.20 ± 0.06^a^	9.72 ± 0.06^a^

Titratable acidity was measured as citric acid, reducing sugars were measured as glucose, and volatile acids were measured as acetic acid. Data were analysed using one-way ANOVA and significant differences were determined by Duncan test (*p* < 0.05). Values are mean ± standard deviation; lowercase letters (a–d) in each column indicate the least significant difference test, and different letters represent significant differences (*p* < 0.05, Duncan test) between different turbidity of the same kiwifruit variety.

Among the tested varieties, ‘Hayward’ and ‘Qinmei’ kiwifruits exhibited the highest acidity and their fermented wines maintained high acidity levels (Table 1 and Supplementary Table 1). Differences in acidity were mainly related to variety, which has been confirmed by Zhou et al.^5^. Most fruits contain one or two main organic acids such as malic, citric, and tartaric acids, with specific types and quantities varying according to the fruit variety^23^. Tartaric acid is the principal organic acid in grapes^24^, whereas citric acid comprises the majority of the organic acids (>50%) in kiwifruit^25^. Both acids are major components of titratable acids in fruit wine. The titratable acid content generally exhibits a modest increase following AF owing to yeast metabolism during the process^26^, which aligns with the results of this investigation. Acidity significantly affects the flavour of wine, and consumers may reject fruit wines exhibiting high acidity, despite its positive influence on organoleptic support in the wine^17^. With the exception of the ‘Xuxiang’ and ‘Huayou’ wines, the ammonium content in the other three kiwi wines showed no significant differences across the varying clarification levels (Table 1).

Glycerol has been reported to contribute to a round and smooth taste^27^. In this study, all kiwi wines fermented with muddy juice and pulp, except for the ‘Huayou’ wines, contained significantly more glycerol than those fermented with the clarified juices (100 NTU, 200 NTU, and 300 NTU). Glycerol content increased with increasing turbidity in the kiwi wines fermented with clarified juice, but the differences among groups were not significant (Table 1).

Glycerol is an important by-product of AF, and the differences in glycerol levels among kiwi wines fermented with different clarifications of kiwi juice may result from alterations in the osmotic pressure environment to which yeast cells are exposed due to the clarification treatment. This change forces the yeast to balance the internal and external osmotic pressures by releasing glycerol^28^. Furthermore, because muddy juice and pulp contain a higher amount of particulate matter than clarified kiwifruit juice, the heat generated during fermentation may not dissipate promptly, leading to elevated temperatures compared with those in clarified juice fermentation, which could enhance glycerol production^27^.

The brightness of kiwi wine was strongly associated with the clarity of the kiwi juice, which declined substantially as the turbidity of the juice increased (Table 1). With an increase in turbidity, the lightness parameter (L*) decreased, while the red/green index (a*) and the yellow/blue index (b*) values increased. This caused the yellow and green tones of the wine colour to diminish while enhancing the red tone, which was similar to the browning observed by Krapfenbauer et al.^29^ in apple juice processing. Fruit wines that maintain their original juice colour tend to be more appealing to consumers, indicating that kiwi wines produced from clarified kiwi juice can achieve higher sensory scores.

### Effects of varying clarification levels on the volatile compound profiles of kiwi wine

#### Volatile compounds

**Fig. 1:**
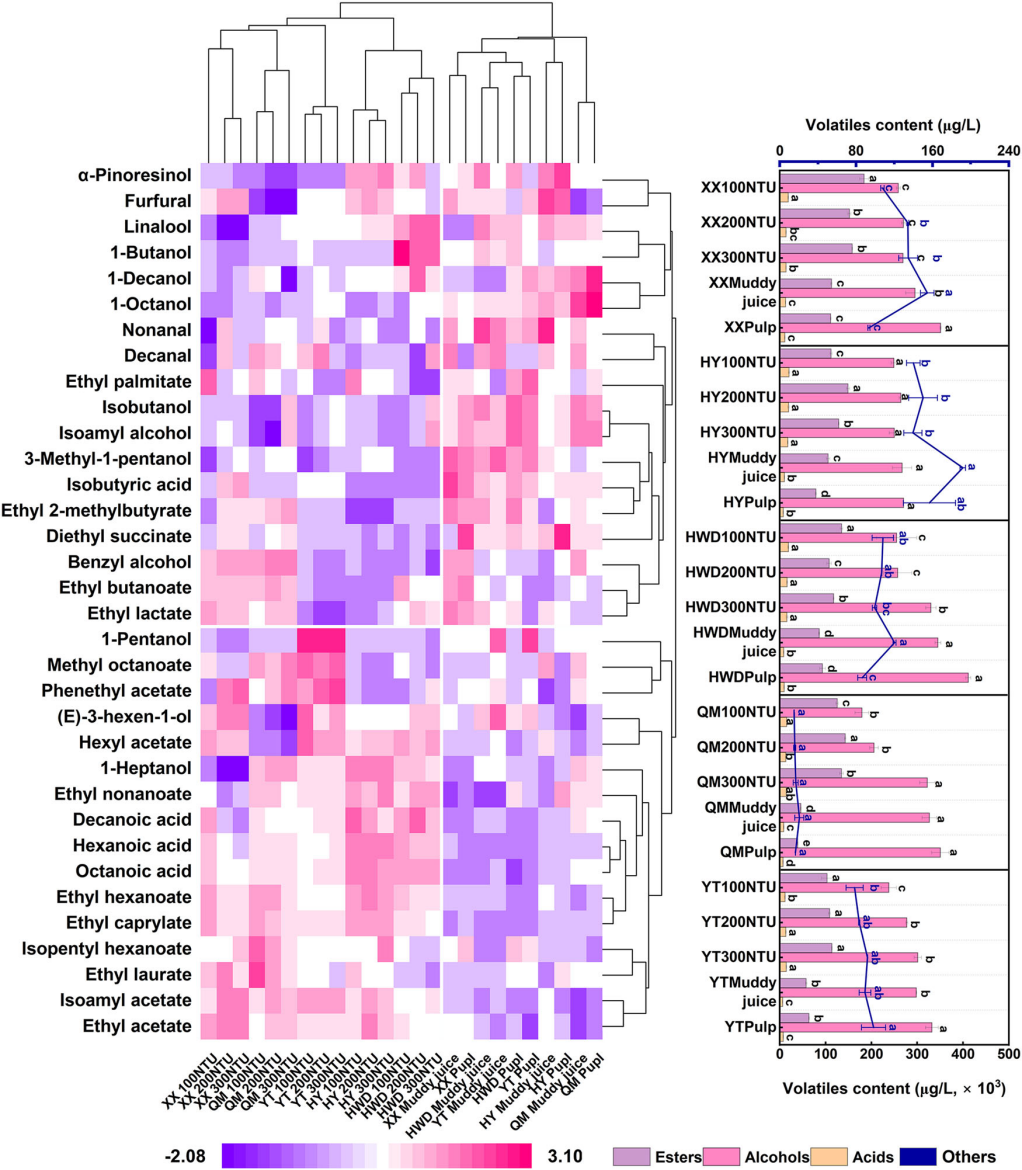
Cluster heat map of volatile compounds. The columns of labels with capital letters represent the kiwi wines fermented from different kiwi varieties (YT Yate, HY Huayou, HWD Haywad, QM Qinmei, XX Xuxiang). Different lowercase letters on the values indicate significant differences between different treatment groups at *p* < 0.05.

A total of 34 aromatic compounds were identified in the kiwi wines, comprising 15 esters, 10 alcohols, four acids, and five aldehydes/terpenes. Detailed data are presented in Supplementary Tables 3–7. Aroma clustering heat maps were created to better understand the distribution of these volatile compounds in kiwi wines (Fig. 1). Cluster heat maps distinctly segregated the samples into two categories: kiwi wines fermented in clarified juice (100 NTU, 200 NTU, and 300 NTU) and kiwi wines fermented in muddy juice and pulp. Overall, this analysis suggests that the clarity of kiwi juice significantly influences the composition and content of aromatic substances in kiwi wines.

Esters contribute significantly to the aromas of fruit wines, primarily by imparting floral and fruity aromas^30^. Ethyl butanoate is the most abundant ester in kiwifruit and is considered to be its core aromatic component^31^. However, isoamyl acetate, ethyl lactate, and ethyl acetate were the main esters in kiwi wine, with their combined contents representing 92.2–96.3% of the total volatile esters. Ethyl acetate was the most abundant, accounting for 61.8–79.2% of the total volatile esters. The contribution of ethyl acetate to wine aromas is double-sided, with low levels bringing a fresh pineapple flavour to wines, while high levels impart a pungent nail polish flavour^32^. The highest level of ethyl acetate in this study was 131.7 mg/L, which is significantly lower than the threshold of 170 mg/L reported in white wines^33^, suggesting a potential positive contribution to kiwifruit wine aroma. Additionally, in all kiwi wines, the acetate content was higher than the ethyl content, and the ratio between them (acetate/ethyl) could be used to assess the organoleptic effect of the esters, with a higher ratio representing richer tropical fruit notes and a lower ratio indicating more tree fruit notes in wines^34^. Moreover, kiwi wines fermented in clarified juice had a higher ester content than those fermented in muddy juice and pulp. The ratio of ester content to total volatile compounds was 26.5–39.9% (mean 32.3%) in kiwi wines fermented with clarified juice compared to only 9.8–27.6% (mean 19.1%) in those fermented with muddy juice and pulp, indicating that greater clarification promotes the accumulation of aromatic ester compounds. In addition, the trends in the content of partial aroma compounds were also correlated with variety and clarification. A minor increase in isoamyl acetate content with increasing turbidity was observed in kiwi wines fermented with ‘Xuxiang’, ‘Huayou’, and ‘Hayward’ clarified juices, while their fatty acid content showed a minor decreasing trend (Supplementary Tables 3–5). Although these changes were not significantly different, the underlying mechanism requires further attention.

Interestingly, there are contradictory reports on the relationship between juice clarity and the aroma of fruit wine. Some studies have suggested that the pre-fermentation clarification process may remove some of the volatile aroma compounds, such as bound terpenes (glycoside forms), or nutrients associated with the synthesis of volatile aroma compounds, such as nitrogen compounds, negatively affecting the aroma of fruit wine^35,36^. Other studies have proposed that the relationship between juice clarification and wine aroma is not linear, and that factors such as fermentation system size, clarifying agent choice and timing, raw material type, and yeast type collectively influence the formation of esters and various volatiles^16,37^. Indeed, the direct adsorption of clarifying agents during clarification results in only a minor reduction in odour-active substances, with most fermentation aroma substances removed by indirect adsorption after binding to proteins^38^. Consequently, clarification during the pre-fermentation period would reduce the protein content, enabling the release of aromatic compounds produced by fermentation, whereas clarification at the end of fermentation might have the opposite effect. In addition, some solid particles attached to kiwifruit muddy juice and pulp in AF may provide competitive substrates or enzyme inhibitors, potentially inhibiting ester synthesis, necessitating further detailed studies^39^.

Alcohols constituted the most abundant group of volatile compounds in kiwi wine, with their ratios ranging from 56.0% to 71.2% (mean 63.8%) in kiwi wines fermented in clarified juice and from 69.7% to 88.3% (mean 78.7%) in those fermented in muddy juice and pulp. Among these, isobutanol and isoamyl alcohol were predominant, with their combined ratios to total alcohols exceeding 88% in all kiwi wines and reaching 94% in wines fermented from muddy juice and pulp.

The variations in isoamyl alcohol and isobutanol content were probably influenced by clarifying agents, which adsorbed some of the nutrients for the yeast or aroma-synthesis precursors through binding by van der Waals forces or hydrogen bonds^16^, resulting in reduced alcohol content. Additionally, certain alcohols are variety-specific, such as 1-pentanol in Yate wines, 1-butanol in ‘Hayward’ wines, benzyl alcohol exclusively in ‘Xuxiang’ and ‘Qinmei’ wines, and 1-heptanol that was uniquely undetectable in ‘Xuxiang’ wines. Furthermore, this variable is consistent with the classification results based on VIP values (Supplementary Fig. 2). Lan et al.^31^ similarly demonstrated the variety-specific in classifying common kiwifruit varieties on the basis of aroma chemistry.

Fatty acids are another important component of the organoleptic properties of fruit wines, contributing to the preservation of the aromatic balance by impeding the hydrolysis of associated aromatic esters^40^. Notably, hexanoic acid, one of the detected fatty acids, is considered responsible for the fatty and soy taste of fruit wines; therefore, excessive production of this substance should be circumvented^41^. Excluding isobutyric acid, the fatty acid content of kiwi wine fermented with clarified kiwi juice was significantly higher than that of kiwi wine fermented with muddy juice and pulp. Moreover, the concentration of medium-chain fatty acids exhibits a positive correlation with their corresponding ethyl ester contents^42^, which is consistent with the findings of this study. Additionally, solid particles in fruit juices suppress fatty acid biosynthesis in yeast cells, necessitating the direct acquisition of fatty acids from the juice^43^. This phenomenon may result in decreased fatty acid content in muddy juice and pulp wines.

Although aldehydes and terpenes constitute a minimal proportion of volatile compounds, their low perception thresholds contribute significantly to the development of floral and fruity aromas in fruit wines^44^. Terpenes, which primarily exist in fruits as free entities or in conjunction with sugars via glycosides, have a muscatel-like aroma and play a vital role in defining the aroma of the fruit variety^45,46^. The terpene content decreased to varying degrees in all of the kiwi wines fermented using clarified juice, similar to the findings of Burin et al.^16^. This reduction may be attributed to the adsorption of aromatic substances by the added clarifying agent^36^ or the adsorption of the β-glucosidase enzyme employed for hydrolysing glycosidic bonds^47^.

#### PCA of the volatile compounds

**Fig. 2:**
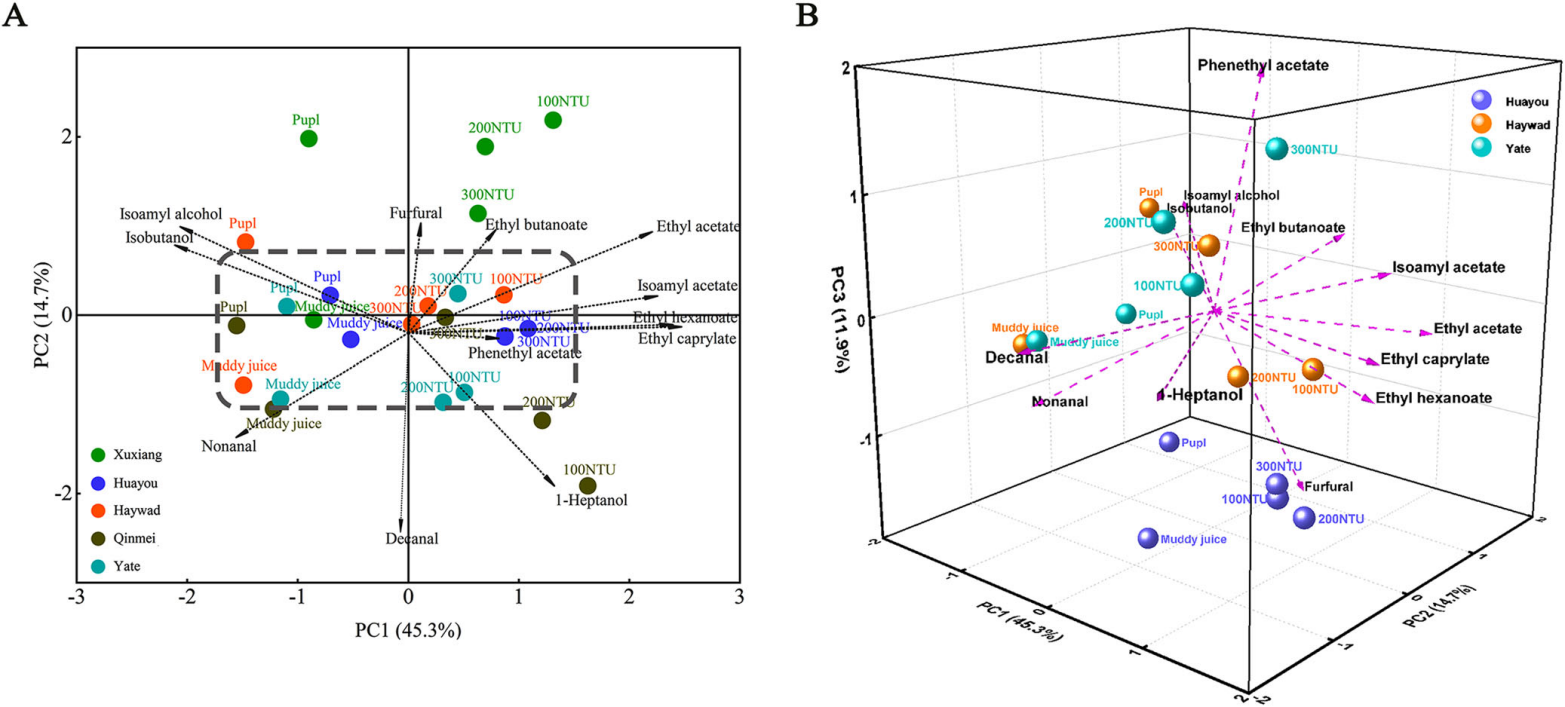
Principal component analysis (PCA) of aroma compounds. **A** PCA of the aroma compounds with OAV > 1 of five kiwifruit wines; **B** Third principal component analysis of aroma compounds with OAV > 1 in “Hayou”, “Haywad”, and “Yate”.

The distribution of volatile compounds in kiwi wine fermented using various kiwi juice clarifications was well-separated in the clustered heat maps (Fig. 1). However, numerous substances exhibited low relative odour activity values (OAV < 0.1), including 1-decanol, 1-octanol, and diethyl succinate, which contribute minimally to the organoleptic aroma. Therefore, to further investigate the compounds that significantly influenced the overall aroma of kiwi wine, we selected only aromatic substances with an OAV > 1 (Table 2) for subsequent PCA (Fig. 2).

**Table 2:** Volatile compounds (μg/L) with OAV > 1 in kiwi wines

Variety	Sample	Isoamyl alcohol	Isobutyl alcohol	1-Heptanol	Isoamyl acetate	Ethyl acetate	Ethyl butanoate	Ethyl hexanoate	Ethyl caprylate	Phenethyl acetate	Decanal	Nonanal	Furfural
Xuxiang	100 NTU	187,463.9 ± 2925.9^c^	70,882.5 ± 3272.8^e^	–	10,035.4 ± 521.5^b^	131,761.09 ± 4924^a^	875.2 ± 44.9^c^	2901.6 ± 169.4^a^	3680.9 ± 181.9^a^	393.6 ± 7.3^e^	2.4 ± 0.8^b^	1.9 ± 0.6^a^	100.1 ± 2.4^b^
	200 NTU	195,600.7 ± 2799.2^c^	73,959.2 ± 1749.4^d^	–	11,205.7 ± 418.1^a^	108,987.5 ± 1294.2^b^	586.0 ± 20.8^d^	1790.5 ± 106.2^b^	2296.9 ± 116.4^b^	2113.4 ± 47.6^b^	4.4 ± 0.3^ab^	4.7 ± 0.2^a^	121.4 ± 0.9^a^
	300 NTU	194,795.8 ± 3227.0^c^	73,654.9 ± 2670.1^c^	–	12,026.9 ± 31.0^a^	113,039.3 ± 178.0^b^	599.6 ± 1.1^d^	1796.3 ± 15.32^b^	2441.2 ± 34.6^b^	2400.7 ± 14.7^a^	3.7 ± 0.0^ab^	3.8 ± 1.2^a^	123.9 ± 8.3^a^
	Muddy juice	213,920.2 ± 14708.7^b^	80,886.0 ± 874.9^b^	–	3982.9 ± 2.4^b^	70,011.4 ± 511.2^c^	965.0 ± 0.4^b^	1116.8 ± 5.6^c^	1079.2 ± 0.4^c^	707.9 ± 26.1^c^	5.9 ± 1.2^a^	5.4 ± 2.0^a^	138.9 ± 8.1^a^
	Pulp	254,287.9 ± 725.7^a^	96,149.6 ± 5522.4^a^	–	4809.8 ± 128.6^b^	70,541.2 ± 16.4^c^	1136.2 ± 20.6^a^	1016.9 ± 56.2^c^	1219.9 ± 63.3^c^	1037.1 ± 7.6^d^	2.1 ± 0.1^b^	3.8 ± 0.9^a^	82.0 ± 0.1^b^
Huayou	100 NTU	170,150.8 ± 4415.8^c^	64,336.3 ± 5.5^d^	14,327.1 ± 123.0^a^	8490.4 ± 194.9^ab^	85,611.2 ± 1200.8^c^	138.9 ± 7.5^bc^	2451.7 ± 74.3^ab^	3034.3 ± 116.4^a^	589.8 ± 25.7^a^	2.9 ± 0.2^a^	3.3 ± 0.1^c^	116.9 ± 4.5^b^
	200 NTU	180,967.9 ± 2634.9^abc^	68,426.3 ± 74.3^c^	14,781.3 ± 108.3^a^	11,226.1 ± 459.4^ab^	118,192.4 ± 1315.3^a^	170.2 ± 9.3^a^	2651.6 ± 104.2^a^	3012.5 ± 115.2^a^	578.2 ± 2.7^a^	3.4 ± 0.8^a^	4.3 ± 0.3^a^	124.5 ± 8.9^ab^
	300 NTU	171,112.2 ± 7773.2^bc^	64,699.8 ± 1186.2^b^	13,706.5 ± 62.9^b^	9262.2 ± 102.1^a^	97,454.2 ± 478.8^b^	131.3 ± 1.1^c^	2255.2 ± 60.6^b^	2834.2 ± 4.5^a^	581.7 ± 17.0^a^	3.3 ± 0.2^a^	3.2 ± 0.0^c^	114.1 ± 5.9^b^
	Muddy juice	192,005.4 ± 15,310.5^ab^	72,599.7 ± 2109.3^a^	2139.1 ± 111.3^c^	6853.9 ± 246.7^ab^	74,263.8 ± 2043.3^d^	157.6 ± 8.6^ab^	1496.4 ± 66.0^c^	1671.9 ± 106.7^b^	309.5 ± 7.0^b^	4.2 ± 0.1^a^	4.1 ± 0.2^ab^	159.2 ± 1.3^a^
	Pulp	194,203.1 ± 524.3^a^	73,430.7 ± 4552.1^a^	2491.5 ± 241.5^c^	3121.5 ± 23.7^b^	56,700.9 ± 847.4^e^	167.3 ± 3.8^a^	1083.9 ± 4.5^d^	1422.8 ± 31.2^b^	187.0 ± 9.8^c^	3.4 ± 0.1^a^	3.6 ± 0.1^bc^	129.6 ± 17.5^ab^
Hayward	100 NTU	174,336.4 ± 31,611.5^c^	65,918.9 ± 202.4^d^	11,110.4 ± 169.7^a^	8394.8 ± 345.1^a^	97,393.4 ± 1637.8^a^	957.0 ± 34.7^a^	2351.2 ± 140.3^a^	2581.3 ± 143.3^a^	671.9 ± 46.1^b^	3.4 ± 1.2^a^	3.2 ± 0.8^a^	75.6 ± 8.0^a^
	200 NTU	178,333.2 ± 21,694.7^c^	67,430.1 ± 4386.8^d^	9074.8 ± 529.0^b^	6407.4 ± 330.5^b^	71,441.1 ± 2769.9^c^	383.3 ± 17.2^b^	1782.6 ± 126.9^b^	2162.3 ± 113.0^b^	721.6 ± 41.8^b^	2.3 ± 0.1^a^	3.7 ± 0.8^a^	73.5 ± 2.5^a^
	300 NTU	231,453.4 ± 8122.4^b^	87,515.6 ± 1841.7^c^	8732.7 ± 137.9^b^	8548.3 ± 68.9^a^	80,085.4 ± 246.5^b^	417.8 ± 5.1^b^	1866.4 ± 30.1^b^	2178.1 ± 3.1^b^	1382.9 ± 101.7^a^	4.7 ± 2.7^a^	3.4 ± 1.1^a^	67.3 ± 0.1^ab^
	Muddy juice	282,226.9 ± 4556.6^a^	106,713.7 ± 7118.2^b^	5318.9 ± 50.7^c^	3677.3 ± 123.2^d^	53,927.2 ± 1300.2^e^	285.9 ± 9.7^c^	988.4 ± 28.6^c^	914.9 ± 28.3^d^	744.2 ± 17.8^b^	5.0 ± 1.6^a^	6.7 ± 2.6^a^	79.1 ± 6.4^a^
	Pulp	294,820.2 ± 4295.4^a^	111,475.4 ± 25517.8^a^	3207.5 ± 134.3^d^	4490.7 ± 324.8^c^	62,209.6 ± 3992.8^d^	363.0 ± 27.0^b^	995.6 ± 69.0^c^	1239.6 ± 37.2^c^	825.4 ± 54.2^b^	3.3 ± 0.0^a^	5.0 ± 2.2^a^	53.7 ± 8.1^b^
Qinmei	100 NTU	120,463.3 ± 12,412.3^b^	45,548.8 ± 2654.5^c^	12,888.8 ± 191.7^a^	8649.8 ± 132.2^c^	85,217.4 ± 1642.0^c^	837.3 ± 25.9^a^	2809.8 ± 58.6^a^	3775.3 ± 26.1^a^	1257.0 ± 31.4^c^	5.9 ± 0.4^a^	3.1 ± 0.5^a^	–
	200 NTU	140,849.5 ± 11,419.3^b^	53,257.0 ± 1915.7^c^	11,470.4 ± 205.8^b^	10,629.5 ± 145.2^a^	100,109.5 ± 511.7^a^	746.6 ± 11.5^b^	2316.8 ± 36.1^b^	2696.1 ± 29.2^b^	1789.8 ± 39.4^b^	4.5 ± 0.4^a^	4.2 ± 0.0^a^	–
	300 NTU	225,828.1 ± 13,780.6^a^	85,388.6 ± 2852.9^d^	10,638.5 ± 85.9^c^	9894.0 ± 309.2^b^	91,657.9 ± 1951.3^b^	662.8 ± 21.0^c^	1902.3 ± 75.2^c^	2337.5 ± 99.1^c^	2186.1 ± 74.3^a^	3.6 ± 1.3^a^	3.9 ± 0.6^a^	–
	Muddy juice	231,493.3 ± 11115.2^a^	87,530.7 ± 1768.1^b^	7212.7 ± 41.2^d^	2550.7 ± 57.0^d^	28,442.6 ± 386.3^d^	174.0 ± 2.3^d^	852.2 ± 20.3^d^	1191.5 ± 27.3^d^	1055.9 ± 3.3^d^	4.2 ± 2.04^a^	4.3 ± 0.9^a^	–
	Pulp	250,988.8 ± 6894.9^a^	949,02.2 ± 690.3^a^	4491.3 ± 66.4^e^	1525.3 ± 37.9^e^	25,651.4 ± 187.9^d^	31.4 ± 1.42^e^	723.1 ± 29.8^d^	926.7 ± 58.1^e^	492.9 ± 13.9^e^	2.9 ± 0.6^a^	3.4 ± 0.2^a^	–
Yate	100 NTU	152,647.6 ± 24,385.8^c^	57,718.1 ± 11,429.4^d^	6713.5 ± 392.6^b^	9755.9 ± 879.0^a^	77,790.6 ± 6283.8^a^	125.4 ± 9.1^b^	1433.2 ± 135.1^a^	2095.8 ± 230.0^a^	1864.2 ± 191.3^c^	4.4 ± 0.1^ab^	3.3 ± 0.2^a^	63.6 ± 5.7^b^
	200 NTU	181,165.9 ± 615.1^bc^	68,501.2 ± 1906.6^c^	6421.5 ± 81.7^b^	10,078.9 ± 224.5^a^	81,229.0 ± 965.6^a^	155.1 ± 4.2^a^	1451.7 ± 37.4^a^	2234.6 ± 54.8^a^	2427.2 ± 10.2^b^	5.2 ± 0.3^a^	4.2 ± 0.3^a^	64.6 ± 0.8^ab^
	300 NTU	197,230.7 ± 9045.9^ab^	74,575.5 ± 2191.3^c^	7451.6 ± 15.5^a^	11,136.3 ± 165.8^a^	84,458.9 ± 119.2^a^	165.7 ± 2.4^a^	1504.3 ± 50.7^a^	2236.8 ± 10.4^a^	2774.2 ± 88.7^a^	3.2 ± 0.5^b^	3.0 ± 1.1^a^	75.0 ± 1.7^ab^
	Muddy juice	205,153.3 ± 3611.9^ab^	77,571.2 ± 2724.0^b^	3007.7 ± 23.5^c^	3770.0 ± 57.3^b^	38,119.8 ± 675.2^b^	78.0 ± 0.34^c^	834.4 ± 9.2^b^	838.1 ± 0.8^b^	823.6 ± 1.56^d^	4.5 ± 0.1^ab^	5.0 ± 0.6^a^	68.0 ± 5.1^ab^
	Pulp	228,331.4 ± 15,769.5^a^	86,335.1 ± 2612.9^a^	3235.5 ± 46.5^c^	4393.3 ± 30.7^b^	43,667.9 ± 49,379^b^	65.3 ± 1.7^c^	983.5 ± 21.9^b^	1143.0 ± 41.2^b^	810.2 ± 19.0^d^	3.1 ± 0.9^a^	4.4 ± 1.5^a^	81.2 ± 6.16^a^

Values are mean ± standard deviation. Different letters in the same column indicate significant differences (*p* < 0.05, Duncan test). “–“ Indicates that this aroma compound was not detected in the kiwi wine.

Kiwifruit wines fermented using various juice clarifications were distinguished within the first two principal components. Moreover, those produced with the “Xuxiang” and “Qinmei” varieties demonstrated notable separation at the PC2 level (Fig. 2A). A subsequent analysis was conducted on the third principal component regarding the wines of “Hayou”, “Haywad”, and “Yate” (Fig. 2B).

Approximately 60% of the variation in the values could be explained by the first two principal components, with PC1 accounting for 45.3% and PC2 accounting for 14.7% of the total variance. For all five kiwifruit varieties, kiwi wine fermented using clarified kiwi juice was distinctly separated from that fermented using muddy juice and pulp along PC1; the former was located at the positive end of PC1, whereas the latter was located at the negative end. Kiwi wines fermented using clarified juice were closely associated with esters, which are known to contribute to various aroma notes such as apple, honey, rose, green apple, banana, pear, strawberry, fruit, and floral scents^48,49^. In contrast, isoamyl alcohol, isobutanol, and nonanal were closer to muddy juice and pulp wines, adding spicy, whisky, cheese, grassy, and herbal flavours^44,50^. Decanal, which is characterised by a strong citrus peel odour, is an essential secondary volatile component in fruit wines^51^. Even at low concentrations, decanal has been shown to positively affect the flavour profile of fruit wines. In this study, PCA revealed a higher correlation between decanal and specific kiwi varieties. Along PC2, wines fermented using the ‘Xuxiang’ kiwifruit variety were substantially different from those fermented using the ‘Qinmei’ and ‘Yate’ varieties, indicating a connection with variety specificity^5^. Although a better distinction was obtained along PC1 between kiwi wines fermented with clarified juice and those fermented with muddy juice and pulp in the Hayward, Yate, and Huayou varieties, the differences between their varieties were not reflected (dashed box). Along PC3, the kiwifruit wine fermented by the Huayou variety was significantly separated from the first two varieties.

### Effects of varying clarification levels on the sensory characteristics of kiwi wine

**Fig. 3:**
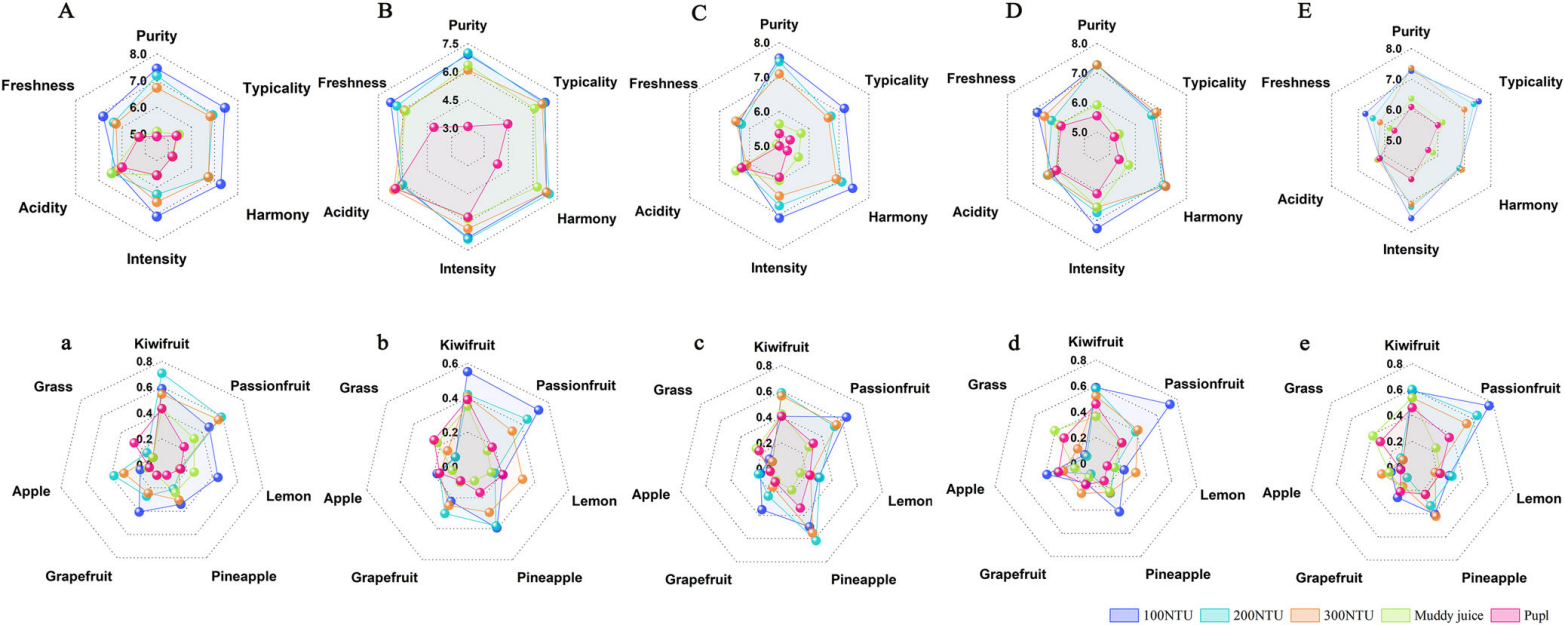
QDA of kiwi wines fermented with different clarifications of kiwifruit juice. The different bold letters represent kiwifruit wines fermented from different kiwifruit varieties (A/a Xuxiang, B/b Huayou, C/c Haywad, D/d Qinmei, E/e Yate).

The sensory characteristics of kiwi wines produced from various types of kiwi juice were assessed by Quantitative descriptive analysis (QDA) (Fig. 3 and Supplementary Data 1). In general, the sensory attributes of kiwi wines produced from clarified kiwi juice exceeded those of wines produced from muddy juice and pulp, exhibiting enhanced purity, typicality, harmony, intensity, and freshness (Fig. 3A–E). This notable difference may be attributed to the higher abundance of fatty acids in clarified kiwi juice (Fig. 1), which is known to greatly influence the sensory characteristics of fruit wines^40^. In contrast, the acidity scores of the groups were similar, which was consistent with the measured titratable acidity distribution of the kiwi wines (Table 1). Although differences in titratable acidity were observed among some of the different clarification groups, the levels of data are close to each other, and no significant differences in acidity scores were observed, suggesting that such differences cannot be perceived in sensory tasting. This observation suggests that the clarification process does not affect the contribution of acidity to the sensory quality of kiwi wines.

Kiwi wines fermented in clarified juice exhibited more kiwifruit, passionfruit, grapefruit, and pineapple aromas than those fermented in muddy juice and pulp, which was consistent with the distribution of aroma compounds in the different kiwi wines (Fig. 1). This was likely attributable to the increased presence of esters and aldehydes in the clarified kiwi juice wines, which also indicated the high tasting accuracy of the panel members. In addition, the flavours of kiwifruit, passionfruit, and pineapple showed high perceived intensity scores for the assessments of all kiwi wines, suggesting that these flavours can be considered typical characteristics of kiwi wines. Kiwi wines produced from muddy juice and pulp presented a grassy odour, which was generally regarded as unfavourable wines, primarily derived from the higher contents of isoamyl alcohol and isobutanol^44^, corresponding to the PCA plot (Fig. 2). Furthermore, these two alcohols have been reported to suppress the perception of fruit-like aromas in wines^52^, potentially contributing to the diminished fruity flavour in wines fermented with muddy juice and pulp. Notably, although no apparent differences were detected between kiwi wines fermented at different levels of clarification (100 NTU, 200 NTU, and 300 NTU) in the aroma compound profiles (Fig. 1 and Supplementary Tables 3–7), the sensory analyses of the kiwi wines fermented with the 100 NTU juice exhibited better freshness, typicality, and intensity, as well as greater passionfruit aroma in the sensory evaluation by the panel, which suggests the need for sensory analyses in the final evaluation of fruit wine quality.

## Materials and methods

### Fermentation yeast strain and kiwifruit varieties

*S. cerevisiae* CEC01, obtained from Angel Yeast Company (Hubei, China), was used for production tests. Five kiwifruit (*Actinidia deliciosa*) varieties, including ‘Xu Xiang’, ‘Huayou’, ‘Hayward’, ‘Qinmei’, and ‘Yate’, were acquired in Yangling, Shaanxi, China. The physicochemical parameters of the raw kiwifruit are shown in Supplementary Table 1.

### Preparation of kiwi must be for clarification treatments

Kiwifruits must be homogenised after peeling; thus, peeled kiwifruit was crushed and thoroughly mixed in a glass tank with the aim of blending the components throughout the fermentation process. After washing, peeling, and homogenising the kiwifruits, the SO_2_ concentration was adjusted to 60 mg/L using H_2_SO_3_ (>99%; Beijing InnoChem Science & Technology Co., Ltd., Beijing, China). After standing for 20 min, the kiwifruit pulp was obtained by adding 20 mg/L pectinase (RF, AB Enzymes GmbH, Germany) for enzymolysis at 20 °C for 12 h. A portion of the kiwifruit pulp was reserved for pulp fermentation and the residual portion was pressed using a vacuum airbag press (bn/qnyz-1m³, Bo Brewing & Wine Technology Service Co., China) to yield muddy kiwifruit juice. A portion of the muddy kiwifruit juice was set aside for muddy juice fermentation and the remaining sample was clarified by adding 0.5 g/L bentonite (FermoBent® PORE-TEC, Germany), followed by storage at 4°C for two days. The clarified kiwifruit juice was separated, and the clarity of the isolated juice was adjusted to 100 NTU, 200 NTU, and 300 NTU using the lower sediment. In summary, five different samples were obtained varying in clarification levels: 100 NTU, 200 NTU, 300 NTU, muddy juice, and fruit pulp.

### Kiwi wine fermentation

*S. cerevisiae* CEC01 was precultured in a YPD liquid medium (10 g/L yeast extract, 20 g/L peptone, and 20 g/L glucose) at 25°C for 48 h before inoculation. Subsequently, the juice was inoculated with the yeast liquid culture at a density of 1 × 10^6^ CFU/mL. Each treatment was conducted in triplicate. Fermentation was performed at 20°C under static conditions, and reducing sugars were monitored daily as described below in “Determination of basic physicochemical parameters”. The fermentation process was terminated by adding 60 mg/L of SO_2_ when the reducing sugar level remained constant for two days. Following fermentation, the kiwi wine was separated into new tanks and stored at 4 °C in a well-ventilated area for three weeks before subsequent analysis.

### Determination of basic physicochemical parameters

The content of reducing sugar was measured according to Kopsahelis et al.^53^ using a high-performance liquid chromatography (LC1260, Agilent, Santa Clara, CA, USA) equipped with an Agilent Hi-Plex H (300 × 7.7 mm; USA) column. Alcohol content and pH were evaluated according to the methods described by Huang et al.^25^. The content of titratable acidity, and volatile acids was measured according to the National Standard of the PRC GB/T 15038-2006 (General Administration of Quality Supervision, Inspection and Quarantine of the PRC, 2006). Titratable acidity was determined using acid-base neutralisation titration. Volatile acids were distilled using water vapour distillation followed by acid-base titration.

Glycerol and ammonium levels were determined using a Y15 automatic wine analyser (Biosystems, Barcelona, Spain) according to the manufacturer’s instructions. CIELab colour parameters were determined according to the literature^54^, and samples were scanned in the visible spectrum (400–700 nm) using an ultraviolet spectrophotometer (UV-2550, Shimadzu, Japan) and 10-mm quartz cuvettes. The parameters L*, a*, and b* represent the brightness, red/green index, and yellow/blue index, respectively.

### Volatile compound analysis

Volatile compounds were analysed using headspace solid-phase microextraction coupled with gas chromatography-mass spectrometry according to the method reported by Chen et al.^55^, with an HP-INNOWAX column (60 m × 0.25 mm × 0.25 μm; Agilent J & W, USA) and a PAL autosampler. Briefly, a 5 mL sample, 10 μL internal standard (4-methyl-2-pentanol, 1.0 g/L), and 1 g NaCl were placed in a 20 mL headspace vial, which was then capped and positioned on the sample tray. The sample vial was placed in a heated kettle and equilibrated at 40 °C for 30 min with a shaking speed of 400 rpm, the extraction head was then inserted into the sample, and the sample was injected after 30 min of adsorption at 40 °C and 250 rpm. High-purity helium was used as the carrier gas at a flow rate of 1 mL/min and the inlet temperature was 250 °C for 25 min. The ramp-up procedure was 50 °C for 1 min and then ramped up to 220 °C at 3 °C/min for 5 min. The mass spectrometry interface temperature was 280 °C, the ion source temperature was 230 °C, the mode was set to electron ionisation with an ion energy of 70 eV, and the mass spectrometry scan range was 29–350 u.

Standard calibration curves were established using volatile compound standards (see Supplementary Table 2) in a synthetic wine medium (11% v/v ethanol, 6 g/L tartaric acid, and pH adjusted to 3.4 with 1 M NaOH) according to the method of Huang et al.^25^. An Agilent ChemStation was used to quantify the volatile compounds. The concentrations of the compounds were then calculated using the calibration curves as described by Chen et al.^55^.

### Sensory assessment

The sensory assessment was performed by a tasting panel as described by Wang et al.^56^. The tasting panel comprised 11 experienced tasters (six males and five females) between the ages of 20 years and 24 years, organised by the College of Oenology, Northwest A&F University, and each participant was given informed consent prior to their participation in the study. Following the methodology described by Bai et al.^57^, the panel was pre-trained in rigorous aroma identification using the learning tool Le Nez du Vin (Masterkit 54; France), which contains 54 common wine aromas. The reliability of the panel was assessed using the *F*-value of the test statistic and the mean-square error value provided by the Panel Check software. Prior to tasting the official sample, an aliquot of commercial kiwi wine was used for familiarisation training. Formal sensory tasting was conducted in a standardised tasting room at the College of Oenology. The overall sensory evaluation was based on a nine-point scale, where 0 represents a deficiency and 9 represents the highest intensity of six attributes: purity, typicality, harmony, intensity, acidity, and freshness. The final score was obtained by calculating the arithmetic mean after excluding the highest and lowest scores.

The QDA of the aroma of kiwifruit wine was conducted using the process shown below. During the sensory evaluation of the Kiwi wine samples, the panel was required to use the descriptors in Le Nez du Vin to characterise the aromas of the samples. To identify the characteristic aromas, evaluators were asked to stand still for 5–7 s, shake the glass, and repeat the sniffing for an additional 8–10 s. Each kiwi wine was evaluated for its characteristic aromas using five aroma descriptors and the aroma strength was recorded on a six-point scale: 0, very weak; 1, weak; 2, slightly weak; 3, medium; 4, slightly strong; and 5, strong. The characteristic intensity of an aroma was calculated as the geometric mean of the frequency of use and quantified intensity of a given aroma term using Eq. (1) :

1 $${\rm{M}}\,=\,\sqrt{F* I}\,\times\,100 \%$$

where *F* is the number of times the descriptor was actually mentioned as a percentage of the total number of times the descriptor could have been mentioned and *I* is the intensity of a descriptor provided by the evaluation panel. The *M*-value represents the quantitative value of a wine’s aroma attributes. All samples were subjected to two replicate tastings using the following process: (i) after randomly ordering the samples, the panel performed the first round of tasting on all of the samples; (ii) a 30-min break was taken; and (iii) after randomly ordering the samples a second time, the panel performed a second round of tasting on all of the samples.

### Statistical analysis

The data were compiled and summarised in Excel 2016 and are expressed as mean ± standard deviation. Differences among groups were evaluated using a one-way analysis of variance in SPSS (v26.0, IBM, USA) with Duncan’s multiple-range test assessed at the α = 0.05 level. PCA was performed using XLSTAT (Addinsoft SARL), with data normalised using the *Z*-score method, to compare the volatile characteristics of wines fermented using the different treatments. Orthogonal partial least-squares discriminant analysis was performed using SIMCA 16.0.1 (Umetrics, Umea, Sweden). Visualizations were prepared using Origin 2023b software (OriginLab, Northampton, MA, USA).

### Supplementary information

Supplementary Information

Supplementary Data 1

## Data Availability

The authors declare that all data supporting the findings of this study are available in the paper and in supplementary information.
