# Supplementary material for: Impact of must clarification treatments on chemical and sensory profiles of kiwifruit wine
Source: NPJ Sci Food. 2024 Jun 25;8:40. doi: 10.1038/s41538-024-00280-z (PMC11199669; doi:10.1038/s41538-024-00280-z)
Supplement: Supplementary file 1 — Supplementary Information [file 41538_2024_280_MOESM1_ESM.pdf]

Supplementary information

Supplementary Discussion

To better identify the differences in the aroma compounds of kiwi wines fermented with different varieties, OPLS-DA was performed with variety as the independent variable and the aroma substance as the dependent variable (Supplementary Fig. 2), including 200 replacement cross-validations to evaluate the model fit.

The ODLS-DA results demonstrated that the intercepts of the R^2 and Q^2 regression lines on the vertical axis were less than 1 and the intersection of the Q^2 regression line with the vertical axis was less than 0, which indicated that the model was not overfitted (Supplementary Fig. 2a)[1, 2], thereby validating the model for the analysis of differences in aroma compounds between different kiwi wines. The different varieties of kiwi wine samples were well differentiated in the score scatter three-dimensional plot of ODLS-DA for aroma compounds (Supplementary Fig. 2b). The 'Xuxiang', 'Yate', and 'Qinmei' wines were clustered separately, whereas 'Huayou' and 'Hayward' wines clustered close together, suggesting similarities in the composition and content of aromatic compounds between these latter two wines. 'Qinmei' wine is located in the first quadrant, close to methyl octanoate and phenethyl acetate and diagonally to α-pinoresinol, indicating that 'Qinmei' has the highest content of methyl octanoate and phenethyl acetate and the lowest content of α-pinoresinol (Supplementary Fig. 2c). 'Yate' wine had the highest content of 1-pentanol, which could potentially contribute to an unpleasant bitter almond odour that should be avoided as much as possible in kiwi wines[3]. 'Huayou' and 'Hayward' are positioned together in the third quadrant close to the fatty acids (octanoic, hexanoic, and decanoic acids) as well as 1-butanol. Fatty acids are important precursors for formation of the ester aroma; thus, the higher fatty acid content in 'Huayou' and 'Hayward' wines implies less accumulation of ester aroma, which is consistent with

the aroma distribution trend (Supplementary Fig. 2c). Hexanoic acid gives kiwi wine a soapy odour, which is considered to be a defective aroma[4]. The effect of 1-butanol on the organoleptic aroma of fruit wines has not been studied extensively to date; however, its potential risk to human health should be of concern owing to its methanol-like biochemical properties[5]. 'Xuxiang' wine is located in the fourth quadrant closest to the ethyl esters (ethyl lactate, ethyl butanoate, and ethyl acetate), which are considered to be the core aroma components of kiwi wines, contributing to the formation of the typical aroma of kiwi wines[6]. Additionally, higher levels of aromatic compounds were observed in the fourth quadrant, indicating that the sensory performance of the 'Xuxiang' wines may be more prominent.

The variable importance in projection (VIP) parameter was calculated to classify the variances and explain the contribution of variables to the model[7], where VIP > 1 indicates that the variable contributes significantly to the classification of the samples in each group and can be used as a marker of variance for the component. The VIP analysis showed that the 12 aroma compounds can be used as markers of differences among kiwi wines produced with five kiwifruit varieties (Supplementary Fig. 2d, red columns).

Supplementary References

1. Du, H. et al. Discrimination of authenticity of *Fritillariae Cirrhosae* Bulbus based on terahertz spectroscopy and chemometric analysis. *Microchem. J.* **168,** 106440 (2021). https://doi.org/10.1016/j.microc.2021.106440

2. Kandasamy, S. et al. 1H HRMAS-NMR based metabolic fingerprints for discrimination of cheeses based on sensory qualities. *Saudi J Biol Sci.* **27,** 1446-1461 (2020). https://doi.org/10.1016/j.sjbs.2020.04.043
3. Fan, X. et al. Characterizing the volatile compounds of different sorghum cultivars by both GC-MS and HS-GC-IMS. *Food Res. Int.* **140,** 109975 (2020). https://doi.org/10.1016/j.foodres.2020.109975.
4. Ye, M., Yue, T., & Yuan, Y. Effects of sequential mixed cultures of *Wickerhamomyces anomalus* and *Saccharomyces cerevisiae* on apple cider fermentation. *FEMS Yeast Res.* **14,** 873-882 (2014). https://doi.org/10.1111/1567-1364.12175
5. Segal, D. et al. Issues in assessing the health risks of n-butanol. *J Appl Toxicol.* **40,** 72-86 (2020). https://doi.org/https://doi.org/10.1002/jat.3820
6. Lan, T. et al. Analysis of the aroma chemical composition of commonly planted kiwifruit cultivars in China. *Foods.* **10,** 1645 (2021). https://doi.org/10.3390/foods10071645
7. Arendse, E. et al. Evaluation of biochemical markers associated with the development of husk scald and the use of diffuse reflectance NIR spectroscopy to predict husk scald in pomegranate fruit. *Sci. Hortic.* **232,** 240-249 (2018). https://doi.org/10.1016/j.scienta.2018.01.022

Supplementary Figure 1 Fermentation curves for kiwi wine fermented with different kiwifruit varieties

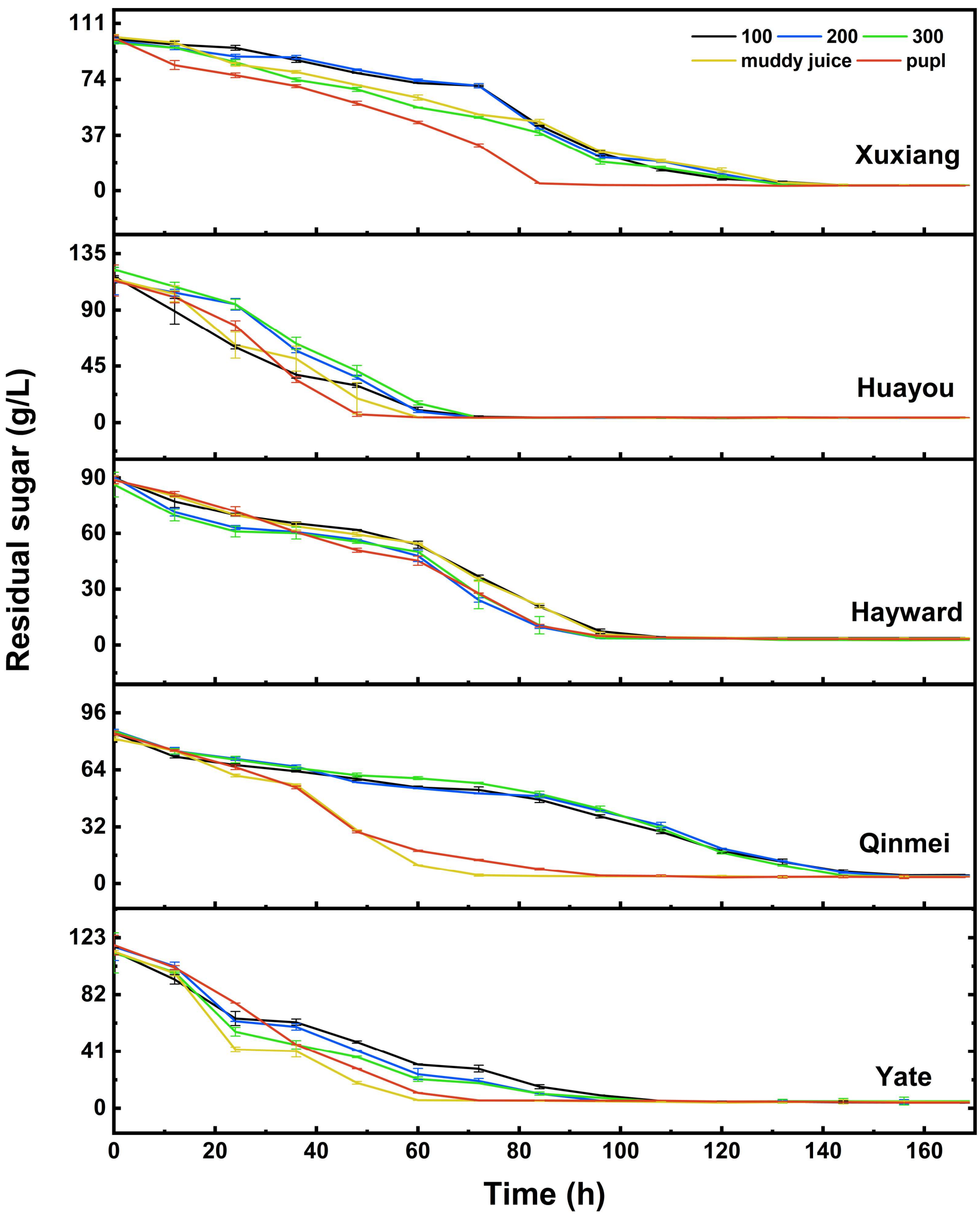

Supplementary Figure 2 Orthogonal partial least-squares discriminant analysis (OPLS-DA) of aroma compounds in kiwi wine fermented with different kiwifruit varieties

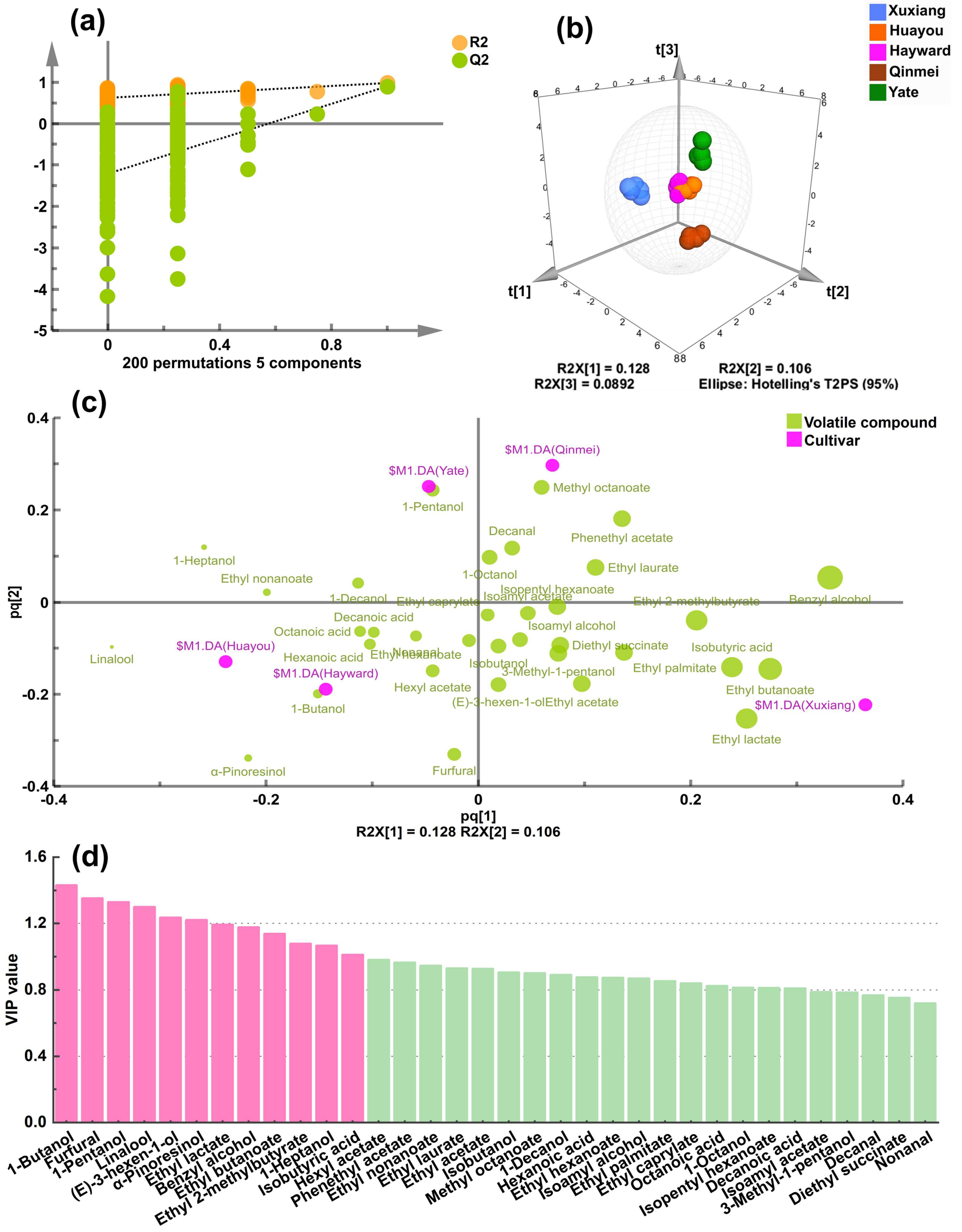

Note: Cross-validation of OPLS-DA model (a), score scatter three-dimensional plot (b), OPLS loading plot (c), VIP (Variable Importance for the Projection) plot (d). The black ellipse represents 95% of the variability.

Supplementary table 1. Physicochemical parameters of kiwifruit

Cultivars	Reducing sugar (g/L)	Titratable acidity (g/L)	Ammonium (mg/L)
Xuxiang	101.60 ± 1.52^{ab}	13.31 ± 0.45^{b}	31.25 ± 1.77^{d}
Huayou	113.52 ± 12.33^{a}	13.24 ± 0.99^{b}	57.45 ± 1.77^{b}
Hayward	88.79 ± 1.90^{b}	16.05 ± 1.25^{a}	58.90 ± 1.84^{b}
Qinmei	84.55 ± 1.22^{b}	16.91 ± 1.23^{a}	126.00 ± 2.26^{a}
Yate	117.61 ± 7.11^{a}	14.04 ± 1.04^{ab}	38.40 ± 2.55^{c}

Note: Reducing sugars were measured as glucose and titratable acidity as citric acid. Values are mean ± SD; lowercase letters (a-d) in each column indicate the least significant difference test, and different letters represent significant differences ($p < 0.05$, Duncan test) between different turbidity of the same kiwifruit variety.

Supplementary table 2. Standard curves of volatile compounds

Retention time (min)	Compounds	Equation	R^2	Dynamic range (μg/L)
20.9	ethyl lactate	y=0.228237x - 0.009666	0.9993	57.4–29380.0
6.29	ethyl acetate	y=0.084566x	0.9983	764.5–391400.1
21.43	rosenoxide	y=167.440807x + 0.022709	0.9992	1.1–535.5
36.18	3-methylthiopropanol	y=0.067235x - 5.661590E - 005	0.9961	20.5–10500.0
30.53	2,3-butanediol	y=0.052458x - 0.180648	0.9924	978.5–501000.2
7.68	ethyl isobutyrate	y=17.862023x - 0.027499	0.9972	0.4–222.5
31.75	terpinen-4-ol	y=15.385727x - 0.008082	0.9992	0.5–275.8
42.58	methyl octalactone	y=2.585884x - 0.059142	0.9982	9.9–5110.2
43.35	phenylethyl alcohol	y=0.777174x + 2.474918	0.9913	1955.1–1001000.0
8.77	isobutyl acetate	y=9.060944x - 0.009300	0.9985	0.3–174.8
28.64	2-methoxy-3-isobutylpyrazine	y=68.291032x - 0.017694	0.9993	0.8–416.5
9.41	n-propanol	y=0.009825x - 0.188170	0.9992	15746.1–8062000.0
33.32	decanoic acid, ethyl ester	y=56.184171x + 1.964255	0.9978	41.9–21460.0
9.82	ethyl 2-methylbutyrate	y=8.183730x - 0.004598	0.9986	0.3–172.8
10.31	ethyl isovalerate	y=23.554194x + 0.007214	0.999	0.3–174.0
26.31	Furfural	y=4.458297x + 0.011912	0.9992	4.6–2346.5
10.89	isobutanol	y=0.092808x - 0.061664	0.9988	378.1–193600.2
40.7	hexanoic acid	y=1.308639x - 0.149991	0.9982	41.1–21020.3
12.14	isoamyl acetate	y=8.723264x + 0.127365	0.9991	15.8–8094.2
25.54	heptanol	y=5.119414x + 0.010468	0.9979	3.1–1534.2
7.94	2,3-Butanedione	y=0.121182x - 0.215280	0.9888	153.6–78670.0
15.03	limonene	y=42.069443x - 0.082935	0.9661	0.2–124.3
15.28	isoamyl alcohol	y=0.147323x + 0.725713	0.9975	1940.0–993300.4
16.59	ethyl hexanoate	y=19.652581x	0.984	45.6–23350.1
17.05	1-pentanol	y=0.154690x + 0.743608	0.9969	1545.3–791200.1
17.99	hexyl acetate	y=44.145605x + 0.048898	0.9992	0.8–400.3
18.73	acetoin	y=0.011637x - 0.001574	0.9985	189.4–96950.0
18.74	n-octanal	y=5.052631x - 0.002345	0.998	0.3–144.2
19.56	1-pentanol, 4-methyl-	y=1.697774x - 0.005225	0.9991	6.2–3175.1
20.12	1-pentanol, 3-methyl-	y=2.105434x - 0.012673	0.9995	23.2–11860.2
21.22	1-hexanol	y=2.622024x + 0.128568	0.999	33.0–16900.5

Retention time (min)	Compounds	Equation	R^2	Dynamic range (μg/L)
21.7	3-hexen-1-ol, (E)-	y=0.595736x - 6.680709E - 004	0.9988	3.2–1623.0
22.61	3-hexen-1-ol, (Z)-	y=0.800709x - 6.392975E - 005	0.999	3.3–1679.0
22.92	methyl octanoate	y=123.348550x - 0.048745	0.9996	0.3–175.2
23.15	nonanal	y=13.638923x + 0.012078	0.9918	0.2–102.1
23.91	2-hexen-1-ol, (Z)-	y=1.826203x + 0.052100	0.999	33.6–17190.0
25.1	ethyl caprylate	y=27.395869x - 0.807793	0.9987	20.6–10540.0
25.34	1-octen-3-ol	y=1149.854159x - 0.217900	0.999	0.3–155.9
25.46	acetic acid	y=0.070009x - 0.074902	0.9976	680.3–348300.0
25.83	isopentyl hexanoate	y=130.644268x - 0.112538	0.9963	0.3–171.1
26.97	1-hexanol, 2-ethyl-	y=20.850858x + 0.049861	0.9989	4.1–2076.5
27.55	decanal	y=18.952046x - 0.005246	0.9961	0.3–167.5
28.97	nonanoic acid, ethyl ester	y=49.984072x - 0.062869	0.996	0.4–211.0
29.34	linalool	y=15.616257x - 0.003206	0.9982	0.8–404.2
29.73	1-octanol	y=10.701374x + 0.003248	0.9993	2.2–1133.0
30.24	propanoic acid, 2-methyl-	y=0.061796x - 0.093681	0.9943	393.8–201600.1
34.29	isovaleric acid	y=0.419433x - 0.075127	0.9922	38.0–19470.0
34.65	butanedioic acid, diethyl ester	y=1.855625x - 0.072320	0.9994	79.1–40480.0
37.62	geranyl acetate	y=179.630713x - 0.774457	0.997	1.8–904.1
37.8	citronellol	y=32.677175x - 0.037990	0.9976	0.7–344.0
38.65	methyl salicylate	y=45.660356x - 0.078675	0.9949	0.5–239.2
39.13	neryl alcohol	y=9.662038x - 0.013167	0.9954	0.5–239.0
39.87	ethyl salicylate	y=151.470695x - 0.219330	0.996	0.4–223.9
39.97	phenethyl acetate	y=27.452731x - 0.098430	0.9992	15.9–8150.0
40.7	dodecanoic acid, ethyl ester	y=41.879870x - 0.192895	0.9526	3.3–1698.0
46.41	Phenol	y=1.972829x + 5.513856E - 004	0.999	2.0–1040.0
47.24	phenol, 4-ethyl-2-methoxy-	y=8.045014x - 0.220153	0.9973	8.5–4360.0
47.96	octanoic acid	y=5.817575x - 0.512256	0.998	36.4–18630.1
51.77	phenol, 4-ethyl-	y=6.754569x - 0.055160	0.9916	1.8–906.0
53.94	ethyl hexadecanoate	y=5.486319x + 0.021100	0.99	1.0–504.6
54.46	n-decanoic acid	y=4.243450x - 0.086162	0.9925	5.9–3038.0
56.9	farnesol	y=5.793382x - 0.143558	0.9713	3.5–1796.5
59.38	benzoic acid	y=0.236936x + 0.001323	0.9713	3.1–1579.0

Retention time (min)	Compounds	Equation	R^2	Dynamic range (μg/L)
20.95	ethyl lactate	y=0.228237x - 0.009666	0.9993	57.8–29380.0
6.29	ethyl acetate	y=0.084566x	0.9983	764.4–391400.1
21.43	rosenoxide	y=167.440807x + 0.022709	0.9992	1.1–535.5
36.18	3-methylthiopropanol	y=0.067235x - 5.661590E - 005	0.9961	20.5–10500.1
30.53	2,3-butanediol	y=0.052458x - 0.180648	0.9924	978.5–501000.0
7.68	ethyl isobutyrate	y=17.862023x - 0.027499	0.9972	0.4–222.5
31.75	terpinen-4-ol	y=15.385727x - 0.008082	0.9992	0.5–275.8
42.58	methyl octalactone	y=2.585884x - 0.059142	0.9982	9.9–5110.1
43.35	phenylethyl alcohol	y=0.777174x + 2.474918	0.9913	1955.0–1001000.1
8.77	isobutyl acetate	y=9.060944x - 0.009300	0.9985	0.4–174.8
28.64	2-methoxy-3-isobutylpyrazine	y=68.291032x - 0.017694	0.9993	0.8–416.5
9.41	n-propanol	y=0.009825x - 0.188170	0.9992	15746.1–8062000.3
33.32	decanoic acid, ethyl ester	y=56.184171x + 1.964255	0.9978	41.9–21460.0
9.82	ethyl 2-methylbutyrate	y=8.183730x - 0.004598	0.9986	0.3–172.8
10.31	ethyl isovalerate	y=23.554194x + 0.007214	0.999	0.3–174.2
26.31	Furfural	y=4.458297x + 0.011912	0.9992	4.6–2346.0
10.89	isobutanol	y=0.092808x - 0.061664	0.9988	378.3–193600.4
40.7	hexanoic acid	y=1.308639x - 0.149991	0.9982	41.5–21020.2
12.14	isoamyl acetate	y=8.723264x + 0.127365	0.9991	15.8–8094.0
25.54	heptanol	y=5.119414x + 0.010468	0.9979	3.1–1534.7
7.94	2,3-Butanedione	y=0.121182x - 0.215280	0.9888	153.4–78670.0
15.03	limonene	y=42.069443x - 0.082935	0.9661	0.2–124.3
15.28	isoamyl alcohol	y=0.147323x + 0.725713	0.9975	1940.4–993300.0
16.59	ethyl hexanoate	y=19.652581x	0.984	45.6–23350.0
17.05	1-pentanol	y=0.154690x + 0.743608	0.9969	1545.3–791200.0
17.99	hexyl acetate	y=44.145605x + 0.048898	0.9992	0.8–400.3
18.73	acetoin	y=0.011637x - 0.001574	0.9985	189.4–96950.1
18.74	n-octanal	y=5.052631x - 0.002345	0.998	0.3–144.2
19.56	1-pentanol, 4-methyl-	y=1.697774x - 0.005225	0.9991	6.2–3175.0
20.12	1-pentanol, 3-methyl-	y=2.105434x - 0.012673	0.9995	23.2–11860.5
21.22	1-hexanol	y=2.622024x + 0.128568	0.999	33.0–16900.6
21.7	3-hexen-1-ol, (E)-	y=0.595736x - 6.680709E - 004	0.9988	3.1–1623.0

Retention time (min)	Compounds	Equation	R^2	Dynamic range (μg/L)
22.61	3-hexen-1-ol, (Z)-	y=0.800709x - 6.392975E - 005	0.999	3.3–1679.6
22.92	methyl octanoate	y=123.348550x - 0.048745	0.9996	0.3–175.2
23.15	nonanal	y=13.638923x + 0.012078	0.9918	0.2–102.1
23.91	2-hexen-1-ol, (Z)-	y=1.826203x + 0.052100	0.999	33.5–17190.0
25.1	ethyl caprylate	y=27.395869x - 0.807793	0.9987	20.6–10540.9
25.34	1-octen-3-ol	y=1149.854159x - 0.217900	0.999	0.3–155.9
25.46	acetic acid	y=0.070009x - 0.074902	0.9976	680.3–348300.8
25.83	isopentyl hexanoate	y=130.644268x - 0.112538	0.9963	0.3–171.1
26.97	1-hexanol, 2-ethyl-	y=20.850858x + 0.049861	0.9989	4.1–2076.5
27.55	decanal	y=18.952046x - 0.005246	0.9961	0.3–167.5
28.97	nonanoic acid, ethyl ester	y=49.984072x - 0.062869	0.996	0.4–211.0
29.34	linalool	y=15.616257x - 0.003206	0.9982	0.8–404.2
29.73	1-octanol	y=10.701374x + 0.003248	0.9993	2.2–1133.1
30.24	propanoic acid, 2-methyl-	y=0.061796x - 0.093681	0.9943	393.8–201600.1
34.29	isovaleric acid	y=0.419433x - 0.075127	0.9922	38.0–19470.0
34.65	butanedioic acid, diethyl ester	y=1.855625x - 0.072320	0.9994	79.1–40480.8
37.62	geranyl acetate	y=179.630713x - 0.774457	0.997	1.8–904.0
37.8	citronellol	y=32.677175x - 0.037990	0.9976	0.7–344.0
38.65	methyl salicylate	y=45.660356x - 0.078675	0.9949	0.5–239.2
39.13	neryl alcohol	y=9.662038x - 0.013167	0.9954	0.5–239.9
39.87	ethyl salicylate	y=151.470695x - 0.219330	0.996	0.4–223.9
39.97	phenethyl acetate	y=27.452731x - 0.098430	0.9992	15.9–8150.8
40.7	dodecanoic acid, ethyl ester	y=41.879870x - 0.192895	0.9526	3.3–1698.1
46.41	Phenol	y=1.972829x + 5.513856E - 004	0.999	2.3–1040.3
47.24	phenol, 4-ethyl-2-methoxy-	y=8.045014x - 0.220153	0.9973	8.5–4360.0
47.96	octanoic acid	y=5.817575x - 0.512256	0.998	36.4–18630.0
51.77	phenol, 4-ethyl-	y=6.754569x - 0.055160	0.9916	1.7–906.1
53.94	ethyl hexadecanoate	y=5.486319x + 0.021100	0.99	0.9–504.6
54.46	n-decanoic acid	y=4.243450x - 0.086162	0.9925	5.9–3038.7
56.9	farnesol	y=5.793382x - 0.143558	0.9713	3.5–1796.5
59.38	benzoic acid	y=0.236936x + 0.001323	0.9713	3.1–1579.0

Note: The dynamic range indicates the range of maximum and minimum concentration values of the generated standard curve.

Supplementary table 3. Volatile components in kiwi wine fermented with different clarified juices of the "Xuxiang" variety (μg/L)

Volatile compounds	100NTU	OAV	200NTU	OAV	300NTU	OAV	Muddy juice	OAV	Pulp	OAV
Isoamyl alcohol	187463.84 ± 2925.89[c]	> 1	195600.73 ± 2799.18[c]	> 1	194795.82 ± 3227.02[c]	> 1	213920.15 ± 14708.68[b]	> 1	254287.90 ± 725.72[a]	> 1
Isobutanol	70882.54 ± 3272.78[e]	> 0.1	73959.21 ± 1749.40[d]	> 0.1	73654.86 ± 2670.14[c]	> 0.1	80886.02 ± 874.86[b]	> 0.1	96149.61 ± 5522.37[a]	> 1
1-Decanol	7.70 ± 0.51[ab]	< 0.1	6.80 ± 0.24[ab]	< 0.1	8.06 ± 0.93[a]	< 0.1	5.75 ± 0.40[b]	< 0.1	6.19 ± 0.06[ab]	< 0.1
1-Octanol	27.55 ± 1.07[e]	< 0.1	42.52 ± 2.01[d]	< 0.1	49.42 ± 0.24[c]	< 0.1	73.96 ± 1.01[a]	< 0.1	57.88 ± 0.53[b]	< 0.1
3-Methyl-1-pentanol	67.14 ± 1.43[e]	< 0.1	95.03 ± 1.28[d]	> 0.1	105.20 ± 1.34[c]	> 0.1	157.81 ± 1.03[a]	> 0.1	144.26 ± 2.58[b]	> 0.1
(E)-3-hexen-1-ol	262.72 ± 12.94[a]	> 1	248.3 ± 1.42[a]	> 1	251.56 ± 0.57[a]	> 1	111.40 ± 4.89[b]	> 0.1	86.18 ± 0.68[c]	> 0.1
Isoamyl acetate	10035.38 ± 521.52[b]	> 1	11205.69 ± 418.07[a]	> 1	12026.91 ± 30.98[a]	> 1	3982.94 ± 2.39[b]	> 1	4809.81 ± 128.64[b]	> 1
Hexyl acetate	783.26 ± 46.59[a]	> 1	579.32 ± 31.18[b]	> 0.1	589.96 ± 1.03[b]	> 0.1	113.96 ± 0.66[c]	> 0.1	71.21 ± 3.61[c]	> 0.1
Ethyl acetate	131761.09 ± 4924[a]	> 1	108987.46 ± 1294.15[b]	> 1	113039.27 ± 178.02[b]	> 1	70011.4 ± 511.16[c]	> 1	70541.23 ± 16.35[c]	> 1
Ethyl hexanoate	2901.55 ± 169.4[a]	> 1	1790.46 ± 106.15[b]	> 1	1796.30 ± 15.32[b]	> 1	1116.74 ± 5.56[c]	> 1	1016.91 ± 56.21[c]	> 1
Ethyl lactate	33133.31 ± 983.67[a]	> 0.1	23990.02 ± 171.84[c]	> 0.1	24811.35 ± 61.85[c]	> 0.1	34778.74 ± 669.49[a]	> 0.1	30409.87 ± 171.04[b]	> 0.1
Ethyl caprylate	3680.95 ± 181.87[a]	> 1	2296.89 ± 116.39[b]	> 1	2441.20 ± 34.55[b]	> 1	1079.22 ± 0.41[c]	> 1	1219.93 ± 63.34[c]	> 1
Ethyl nonanoate	2.89 ± 0.15[a]	> 0.1	2.07 ± 0.09[b]	> 0.1	2.51 ± 0.33[ab]	> 0.1	0.56 ± 0.04[c]	< 0.1	0.82 ± 0.27[c]	< 0.1
Ethyl butanoate	875.15 ± 44.93[c]	> 1	586.03 ± 20.75[d]	> 0.1	599.6 ± 1.09[d]	> 1	965 ± 0.44[b]	> 1	1136.22 ± 20.63[a]	> 1
Diethyl succinate	123.12 ± 8.35[c]	< 0.1	296.41 ± 37.11[b]	< 0.1	282.85 ± 39.97[b]	< 0.1	763.17 ± 11.09[a]	< 0.1	789.59 ± 7.82[a]	> 0.1
Ethyl laurate	61.54 ± 0.76[a]	> 0.1	26.52 ± 0.72[c]	> 0.1	41.15 ± 4.62[d]	< 0.1	14.04 ± 0.14[d]	< 0.1	18.68 ± 1.28[cd]	< 0.1
Ethyl palmitate	141.92 ± 1.67[a]	> 0.1	63.58 ± 0.29[b]	> 0.1	71.23 ± 8.64[b]	< 0.1	77.05 ± 8.16[b]	< 0.1	82.8 ± 16.41[b]	< 0.1
Ethyl 2-methylbutyrate	5.53 ± 0.12[d]	< 0.1	13.36 ± 1.07[c]	< 0.1	15.06 ± 0[c]	> 0.1	28.5 ± 1.75[b]	> 0.1	23.58 ± 0.63[a]	> 0.1

Volatile compounds	100NTU	OAV	200NTU	OAV	300NTU	OAV	Muddy juice	OAV	Pulp	OAV
Methyl octanoate	12.17 ± 0.76[a]	> 0.1	7.73 ± 0.46[b]	> 0.1	6.56 ± 0.04[b]	> 0.1	4.08 ± 0.02[c]	> 0.1	3.06 ± 0.14[c]	> 0.1
Isopentyl hexanoate	1.12 ± 0.01[b]	< 0.1	1.09 ± 0.06[ab]	< 0.1	1.24 ± 0.06[a]	< 0.1	1.03 ± 0.04[b]	< 0.1	1.28 ± 0.07[a]	< 0.1
Isobutyric acid	2257.89 ± 36.23[e]	> 0.1	4260.18 ± 4.69[d]	> 0.1	4794.92 ± 3.99[c]	> 0.1	8365.98 ± 21.23[a]	> 0.1	7314.01 ± 250.19[b]	> 0.1
Hexanoic acid	6785.66 ± 127.98[a]	> 0.1	4195.83 ± 165.91[b]	> 0.1	4074.8 ± 19.6[b]	> 0.1	2366.07 ± 85.2[c]	> 0.1	2047.34 ± 2.12[c]	> 0.1
Octanoic acid	7832.67 ± 212.62[a]	> 0.1	4948.6 ± 269.06[b]	> 0.1	4677.87 ± 1.17[b]	> 0.1	1896.79 ± 99.22[c]	> 0.1	1467.31 ± 35.38[c]	> 0.1
Decanoic acid	1395.47 ± 32.98[a]	< 0.1	452.72 ± 29.81[b]	< 0.1	436.36 ± 36.05[b]	< 0.1	147.22 ± 22.5[c]	< 0.1	143.37 ± 0.2[c]	< 0.1
Decanal	2.35 ± 0.77[b]	> 1	4.38 ± 0.31[ab]	> 1	3.69 ± 0.02[ab]	> 1	5.86 ± 1.2[a]	> 1	2.09 ± 0.1[b]	> 1
Nonanal	1.86 ± 0.55[a]	> 0.1	4.71 ± 0.22[a]	> 0.1	3.76 ± 1.18[a]	> 1	5.39 ± 1.96[a]	> 1	3.83 ± 0.91[a]	> 1
Benzyl alcohol	103.1 ± 3.1[b]	< 0.1	93.85 ± 2.3[b]	< 0.1	98.99 ± 1.51[b]	< 0.1	116.87 ± 3.96[a]	< 0.1	126.71 ± 2.46[a]	< 0.1
Phenethyl acetate	393.55 ± 7.32[e]	> 1	2113.37 ± 47.6[b]	> 1	2400.74 ± 14.65[a]	> 1	707.79 ± 26.08[c]	> 1	1037.06 ± 7.62[d]	> 1
α-Pinoresinol	3.26 ± 0.23[c]	-	3.63 ± 0.23[c]	-	3.27 ± 0.06[c]	-	4.43 ± 0.04[b]	-	5.52 ± 0.19[a]	-
Furfural	100.1 ± 2.35[b]	> 1	121.41 ± 0.88[a]	> 1	123.87 ± 8.25[a]	> 1	138.89 ± 8.12[a]	> 1	81.98 ± 0.1[b]	> 1

Note: Values are mean ± standard deviation. Different lowercase letters in the same row indicate significant differences ($p < 0.05$, Duncan test). "-" indicates that the reference threshold was not found for this aroma component.

Supplementary table 4. Volatile components in kiwi wine fermented with different clarified juices of the "Huayou" variety (μg/L)

Volatile compounds	100NTU	OAV	200NTU	OAV	300NTU	OAV	Muddy juice	OAV	Pulp	OAV
Isoamyl alcohol	170150.84 ± 4415.76[c]	> 1	180967.89 ± 2634.85[abc]	> 1	171112.24 ± 7773.2[bc]	> 1	192005.41 ± 15310.54[ab]	> 1	194203.09 ± 524.3[a]	> 1
Isobutanol	64336.27 ± 5.51[d]	< 0.1	68426.34 ± 74.26[c]	< 0.1	64699.79 ± 1186.16[b]	< 0.1	72599.77 ± 2109.25[a]	< 0.1	73430.74 ± 4552.08[a]	< 0.1
1-Decanol	8.08 ± 0.27[b]	< 0.1	8.92 ± 0.55[b]	< 0.1	8.74 ± 0.02[b]	< 0.1	10.68 ± 0.4[a]	< 0.1	10.61 ± 0.53[a]	< 0.1
1-Octanol	27.51 ± 0.85[e]	< 0.1	36.37 ± 1.13[d]	< 0.1	40.79 ± 0.18[c]	< 0.1	89.98 ± 1.26[b]	< 0.1	68.15 ± 1.36[a]	> 1
1-Heptanol	14327.06 ± 123.04[a]	> 1	14781.33 ± 108.32[a]	> 1	13706.45 ± 62.86[b]	> 1	2139.1 ± 111.33[c]	> 0.1	2491.49 ± 241.5[c]	< 0.1
3-Methyl-1-pentanol	86.17 ± 0.57[c]	< 0.1	101.68 ± 2.08[ab]	> 0.1	100.91 ± 5.52[ab]	> 0.1	107.07 ± 1.29[a]	> 0.1	92.77 ± 1.86[bc]	> 0.1
(E)-3-hexen-1-ol	152.42 ± 7.48[a]	> 0.1	138.58 ± 12.44[a]	> 0.1	136.32 ± 0.64[a]	> 0.1	106.49 ± 23.83[a]	> 0.1	48.21 ± 6.3[b]	> 0.1
Isoamyl acetate	8490.42 ± 194.89[ab]	> 1	11226.05 ± 459.35[ab]	> 1	9262.23 ± 102.06[a]	> 1	6853.88 ± 246.65[ab]	> 1	3121.54 ± 23.68[b]	> 1
Hexyl acetate	470.86 ± 14.17[b]	> 0.1	577.82 ± 19.7[a]	> 0.1	564.19 ± 13.74[a]	> 0.1	443.64 ± 18.13[b]	> 0.1	84.27 ± 1.27[a]	> 0.1
Ethyl acetate	85611.21 ± 1200.79[c]	> 1	118192.44 ± 1315.29[a]	> 1	97454.19 ± 478.78[b]	> 1	74263.79 ± 2043.31[d]	> 1	56700.9 ± 847.43[e]	> 1
Ethyl hexanoate	2451.7 ± 74.28[ab]	> 1	2651.57 ± 104.16[a]	> 1	2255.19 ± 60.55[b]	> 1	1496.35 ± 66.02[c]	> 1	1083.85 ± 4.48[d]	> 1
Ethyl lactate	11061.46 ± 62.4[d]	> 0.1	12514.27 ± 412.12[c]	> 0.1	15544.52 ± 104.16[b]	> 0.1	20043.42 ± 81.34[a]	> 0.1	15401.62 ± 1.76[b]	> 0.1
Ethyl caprylate	3034.3 ± 116.39[a]	> 1	3012.49 ± 115.17[a]	> 1	2834.22 ± 4.48[a]	> 1	1671.86 ± 106.73[b]	> 1	1422.76 ± 31.21[b]	> 1
Ethyl nonanoate	4.52 ± 0.64[a]	> 0.1	3.94 ± 0.24[a]	> 0.1	4.43 ± 0.63[a]	> 0.1	1.94 ± 0.04[a]	> 0.1	3.31 ± 1.59[a]	< 0.1
Ethyl butanoate	138.91 ± 7.54[bc]	> 0.1	170.16 ± 9.26[a]	> 0.1	131.27 ± 1.12[c]	> 0.1	157.55 ± 8.62[ab]	> 0.1	167.28 ± 3.8[a]	> 0.1
Diethyl succinate	118.34 ± 0.32[c]	< 0.1	118.61 ± 8.45[c]	< 0.1	139.7 ± 4.59[c]	< 0.1	324.34 ± 13.9[b]	< 0.1	554.63 ± 14.19[a]	< 0.1
Ethyl laurate	49.37 ± 1.3[a]	> 0.1	35.49 ± 2.36[b]	< 0.1	28.43 ± 1.08[c]	< 0.1	14.06 ± 0.21[d]	< 0.1	25.02 ± 0.28[c]	< 0.1
Ethyl palmitate	106.45 ± 8.74[a]	> 0.1	69.7 ± 12.09[b]	< 0.1	66.8 ± 2.05[b]	< 0.1	66.07 ± 12.79[b]	< 0.1	51.61 ± 8.64[b]	< 0.1

Volatile compounds	100NTU	OAV	200NTU	OAV	300NTU	OAV	Muddy juice	OAV	Pulp	OAV
Ethyl 2-methylbutyrate	1.73 ± 0.17^{d}	< 0.1	2.61 ± 0.3^{cd}	< 0.1	2.83 ± 0.17^{c}	< 0.1	5.77 ± 0.36^{b}	< 0.1	8.33 ± 0.16^{a}	> 0.1
Methyl octanoate	4.82 ± 0.26^{b}	> 0.1	4.91 ± 0.13^{b}	> 0.1	4.36 ± 0.06^{b}	> 0.1	5.65 ± 0.34^{a}	> 0.1	2.37 ± 0.08^{c}	< 0.1
Isopentyl hexanoate	1.01 ± 0.1^{ab}	< 0.1	1.12 ± 0.1^{ab}	< 0.1	1.43 ± 0.37^{a}	< 0.1	0.93 ± 0.13^{ab}	< 0.1	0.78 ± 0.14^{b}	< 0.1
Isobutyric acid	1227.7 ± 54.58^{e}	< 0.1	1601.63 ± 5.08^{d}	< 0.1	1966.44 ± 5.7^{c}	> 0.1	2629.31 ± 9.75^{b}	> 0.1	2720.79 ± 4.58^{a}	> 0.1
Hexanoic acid	7809.64 ± 500.07^{a}	> 0.1	7538.31 ± 578.15^{a}	> 0.1	6851.76 ± 93.04^{a}	> 0.1	3594.76 ± 177.77^{b}	> 0.1	2425.06 ± 82.26^{b}	> 0.1
Octanoic acid	9785.51 ± 538.62^{a}	> 1	8961.94 ± 596.76^{ab}	> 1	7904.03 ± 183.95^{b}	> 0.1	3454.68 ± 132.08^{c}	> 0.1	2257.35 ± 22.18^{c}	> 0.1
Decanoic acid	1452.89 ± 27.05^{a}	< 0.1	1168.35 ± 120.89^{b}	< 0.1	807.45 ± 22.42^{c}	< 0.1	365.71 ± 6.47^{d}	< 0.1	287.14 ± 18.87^{d}	< 0.1
Decanal	2.9 ± 0.21^{a}	> 1	3.42 ± 0.84^{a}	> 1	3.32 ± 0.21^{a}	> 1	4.21 ± 0.12^{a}	> 1	3.38 ± 0.09^{a}	> 1
Nonanal	3.31 ± 0.11^{c}	> 1	4.26 ± 0.25^{a}	> 1	3.22 ± 0.02^{c}	> 1	4.06 ± 0.17^{ab}	> 1	3.58 ± 0.05^{bc}	> 1
Phenethyl acetate	589.82 ± 25.74^{a}	> 1	578.15 ± 2.72^{a}	> 1	581.68 ± 16.97^{a}	> 1	309.49 ± 6.97^{b}	> 1	187 ± 9.83^{c}	> 0.1
Linalool	9.54 ± 0.43^{b}	> 0.1	10.63 ± 0.16^{b}	> 0.1	11.26 ± 0.31^{b}	> 0.1	14.65 ± 0.46^{a}	> 0.1	11.09 ± 1^{b}	> 0.1
α-Pinoresinol	7.31 ± 0.14^{b}	-	7.29 ± 0.46^{b}	-	7.63 ± 0.41^{ab}	-	7.15 ± 0.28^{b}	-	9.16 ± 0.76^{a}	-
Furfural	116.86 ± 4.51^{b}	> 1	124.47 ± 8.92^{ab}	> 1	114.05 ± 5.88^{b}	> 1	159.17 ± 1.33^{a}	> 1	129.64 ± 17.49^{ab}	> 1

Note: Values are mean ± standard deviation. Different lowercase letters in the same row indicate significant differences ($p < 0.05$, Duncan test). "-" indicates that the reference threshold was not found for this aroma component.

Supplementary table 5. Volatile components in kiwi wine fermented with different clarified juices of the "Hayward" variety (μg/L)

Volatile compounds	100NTU	OAV	200NTU	OAV	300NTU	OAV	Muddy juice	OAV	Pulp	OAV
Isoamyl alcohol	174336.41 ± 31611.45[c]	> 1	178333.19 ± 21694.70[c]	> 1	231453.40 ± 8122.42[b]	> 1	282226.94 ± 4556.60[a]	> 1	294820.24 ± 4295.44[a]	> 1
Isobutanol	65918.89 ± 202.39[d]	> 0.1	67430.13 ± 4386.82[d]	> 0.1	87515.58 ± 1841.74[c]	> 1	106713.72 ± 7118.15[b]	> 1	111475.41 ± 25517.82[a]	> 1
1-Butanol	3577.29 ± 11.99[a]	< 0.1	2085.57 ± 132.67[b]	< 0.1	1956.73 ± 22.45[b]	< 0.1	1243.47 ± 42.02[d]	< 0.1	1467.49 ± 120.53[c]	< 0.1
1-Heptanol	11110.4 ± 169.74[a]	> 1	9074.81 ± 529[b]	> 1	8732.74 ± 137.87[b]	> 1	5318.91 ± 50.71[c]	> 1	3207.48 ± 134.28[d]	> 1
1-Octanol	37.98 ± 2.72[c]	< 0.1	55.54 ± 4.11[b]	< 0.1	58.15 ± 3.97[b]	< 0.1	58.23 ± 1.83[b]	< 0.1	78.6 ± 3.56[a]	< 0.1
1-Decanol	9.93 ± 0.47[b]	< 0.1	13.91 ± 0.66[a]	< 0.1	9.96 ± 1.08[b]	< 0.1	4.22 ± 0.04[c]	< 0.1	10.15 ± 0.35[b]	< 0.1
3-Methyl-1-pentanol	88.97 ± 0.88[b]	< 0.1	83.79 ± 0.74[b]	< 0.1	87.5 ± 1.23[b]	< 0.1	127.94 ± 2.29[a]	> 0.1	135.36 ± 7.08[a]	> 0.1
(E)-3-hexen-1-ol	164.25 ± 4.23[c]	> 0.1	198.62 ± 0.9[b]	> 0.1	202.91 ± 6.91[ab]	> 0.1	213.58 ± 2.78[a]	> 0.1	168.18 ± 7.92[c]	> 0.1
Isoamyl acetate	8394.78 ± 345.08[a]	> 1	6407.37 ± 330.45[b]	> 1	8548.31 ± 68.91[a]	> 1	3677.3 ± 123.17[d]	> 1	4490.65 ± 324.79[c]	> 1
Hexyl acetate	718.83 ± 38.24[a]	> 0.1	529.49 ± 37.5[b]	> 0.1	581.01 ± 9.47[b]	> 0.1	140.64 ± 3.96[c]	> 0.1	104.13 ± 7.22[c]	> 0.1
Ethyl acetate	97393.41 ± 1637.84[a]	> 1	71441.11 ± 2769.9[c]	> 1	80085.38 ± 246.49[b]	> 1	53927.15 ± 1300.2[e]	> 1	62209.62 ± 3992.77[d]	> 1
Ethyl hexanoate	2351.19 ± 140.34[a]	> 1	1782.58 ± 126.85[b]	> 1	1866.38 ± 30.05[b]	> 1	988.41 ± 28.64[c]	> 1	995.6 ± 68.95[c]	> 1
Ethyl lactate	21821.51 ± 134.8[b]	> 0.1	24206.47 ± 1349.2[ab]	> 0.1	22444.4 ± 676.11[ab]	> 0.1	25049.06 ± 276.49[a]	> 0.1	22592.84 ± 1674.53[ab]	> 0.1
Ethyl caprylate	2581.26 ± 143.33[a]	> 1	2162.26 ± 112.98[b]	> 1	2178.1 ± 3.08[b]	> 1	914.94 ± 28.34[d]	> 1	1239.61 ± 37.15[c]	> 1
Ethyl nonanoate	3.62 ± 0.47[a]	> 0.1	3.87 ± 0.36[a]	> 0.1	4.06 ± 1[a]	> 0.1	0.69 ± 0.03[b]	< 0.1	3.19 ± 0.08[a]	> 0.1
Ethyl butanoate	956.98 ± 34.71[a]	> 1	383.28 ± 17.21[b]	> 0.1	417.77 ± 5.08[b]	> 0.1	285.68 ± 9.69[c]	> 0.1	362.99 ± 27.02[b]	> 0.1
Diethyl succinate	117.24 ± 12.27[c]	< 0.1	116.03 ± 13.54[c]	< 0.1	135.69 ± 15.68[c]	< 0.1	262.45 ± 10.94[b]	< 0.1	404.03 ± 18.6[a]	< 0.1
Ethyl laurate	24.22 ± 1.52[ab]	< 0.1	19.28 ± 2.56[c]	< 0.1	21.79 ± 1.4[bc]	< 0.1	8.51 ± 0.08[d]	< 0.1	28.48 ± 1.74[a]	< 0.1

Volatile compounds	100NTU	OAV	200NTU	OAV	300NTU	OAV	Muddy juice	OAV	Pulp	OAV
Ethyl palmitate	62.72 ± 2.25^{b}	< 0.1	36.47 ± 7.98^{d}	< 0.1	44.34 ± 0.8^{cd}	< 0.1	54.91 ± 1.96^{bc}	< 0.1	98.97 ± 8.98^{a}	> 0.1
Ethyl 2-methylbutyrate	10.7 ± 1.2^{c}	> 0.1	7.19 ± 0.03^{d}	> 0.1	8.89 ± 0.01^{cd}	> 0.1	19.63 ± 1.21^{b}	> 0.1	26.62 ± 1.82^{a}	> 0.1
Methyl octanoate	10.06 ± 0.68^{a}	< 0.1	6.1 ± 0.37^{b}	< 0.1	5.92 ± 0.06^{b}	< 0.1	3.17 ± 0.09^{d}	< 0.1	4.88 ± 0.29^{c}	< 0.1
Isopentyl hexanoate	1.07 ± 0.1^{b}	< 0.1	0.89 ± 0.01^{c}	< 0.1	1 ± 0.02^{bc}	< 0.1	0.62 ± 0.03^{d}	< 0.1	1.35 ± 0^{a}	< 0.1
Isobutyric acid	1654.48 ± 2.04^{d}	< 0.1	1653.26 ± 11.2^{d}	< 0.1	2318.96 ± 38.4^{c}	> 0.1	4427.09 ± 53.19^{b}	> 0.1	5443.23 ± 212.39^{a}	> 0.1
Hexanoic acid	6928.14 ± 504.62^{a}	> 0.1	5880.46 ± 307.27^{b}	> 0.1	5364.7 ± 498.67^{b}	> 0.1	2223.63 ± 157.37^{c}	> 0.1	2180 ± 104.81^{c}	> 0.1
Octanoic acid	9057.73 ± 640.64^{a}	> 1	7454.58 ± 499^{a}	> 0.1	7286.67 ± 1628.48^{a}	> 0.1	1939.87 ± 134.63^{b}	> 0.1	1642.7 ± 77.53^{b}	> 0.1
Decanoic acid	1160.15 ± 22.^{a}	< 0.1	1373.03 ± 217.09^{a}	< 0.1	855.83 ± 17.51^{b}	< 0.1	188.67 ± 1.16^{c}	< 0.1	199.58 ± 5.7^{c}	< 0.1
Decanal	3.43 ± 1.21^{a}	> 1	2.29 ± 0.05^{a}	> 1	4.69 ± 2.65^{a}	> 1	5 ± 1.62^{a}	> 1	3.25 ± 0.01^{a}	> 1
Nonanal	3.23 ± 0.74^{a}	> 1	3.72 ± 0.73^{a}	> 1	3.42 ± 1.08^{a}	> 1	6.72 ± 2.62^{a}	> 1	4.94 ± 2.21^{a}	> 1
Phenethyl acetate	671.94 ± 46.1^{b}	> 1	721.61 ± 41.82^{b}	> 1	1382.91 ± 101.74^{a}	> 1	744.21 ± 17.82^{b}	> 1	825.36 ± 54.24^{b}	> 1
Linalool	19.82 ± 0.81^{a}	> 0.1	20.4 ± 1.34^{a}	> 0.1	20.08 ± 0.86^{a}	> 0.1	20.51 ± 0.45^{a}	> 0.1	16.71 ± 0.69^{b}	> 0.1
α-Pinoresinol	6.10 ± 0.38^{c}	-	6.93 ± 0.5^{bc}	-	3.94 ± 0.69^{d}	-	8.87 ± 0.18^{a}	-	7.7 ± 0.52^{ab}	-
Furfural	75.47 ± 7.99^{a}	> 1	73.48 ± 2.52^{a}	> 1	67.25 ± 0.12^{ab}	> 1	79.14 ± 6.42^{a}	> 1	53.73 ± 8.1^{b}	> 1

Note: Values are mean ± standard deviation. Different lowercase letters in the same row indicate significant differences ($p < 0.05$, Duncan test). "-" indicates that the reference threshold was not found for this aroma component.

Supplementary table 6. Volatile components in kiwi wine fermented with different clarified juices of the "Qinmei" variety (μg/L)

Volatile compounds	100NTU	OAV	200NTU	OAV	300NTU	OAV	Muddy juice	OAV	Pulp	OAV
Isoamyl alcohol	120463.29 ± 12412.32^{b}	> 1	140849.48 ± 11419.33^{b}	> 1	225828.05 ± 13780.64^{a}	> 1	231493.34 ± 11115.16^{a}	> 1	250988.8 ± 6894.91^{a}	> 1
Isobutanol	45548.75 ± 2654.52^{c}	> 0.1	53257.04 ± 1915.67^{c}	> 0.1	85388.56 ± 2852.87^{d}	> 1	87530.68 ± 1768.09^{b}	> 1	94902.17 ± 690.28^{a}	> 1
1-Heptanol	12888.87 ± 191.74^{a}	> 1	11470.43 ± 205.75^{b}	> 1	10638.52 ± 85.94^{c}	> 1	7212.67 ± 41.19^{d}	> 1	4491.25 ± 66.28^{e}	> 1
1-Octanol	32.55 ± 0.49^{d}	< 0.1	34.09 ± 1.23^{d}	< 0.1	43.24 ± 0.53^{c}	< 0.1	110.38 ± 0.08^{b}	< 0.1	205.56 ± 4.28^{a}	< 0.1
1-Decanol	10.07 ± 0.45^{b}	< 0.1	8.37 ± 0.65^{b}	< 0.1	4.8 ± 3.86^{b}	< 0.1	11.5 ± 0.76^{ab}	< 0.1	17.57 ± 0.93^{a}	< 0.1
3-Methyl-1-pentanol	90.48 ± 8.8^{ab}	< 0.1	104.15 ± 1.83^{a}	> 0.1	102.21 ± 0.96^{a}	> 0.1	80.89 ± 0.02^{b}	< 0.1	86.62 ± 1.19^{b}	< 0.1
(E)-3-hexen-1-ol	25.68 ± 0.6^{a}	> 0.1	34.28 ± 11.7^{a}	> 0.1	37.94 ± 24.3^{a}	> 0.1	12.02 ± 0.05^{a}	< 0.1	20.95 ± 9.66^{a}	< 0.1
Isoamyl acetate	8649.83 ± 132.24^{c}	> 1	10629.46 ± 145.22^{a}	> 1	9893.98 ± 309.16^{b}	> 1	2550.69 ± 56.99^{d}	> 1	1525.27 ± 37.9^{e}	> 1
Hexyl acetate	95.94 ± 1.33^{a}	> 0.1	78.61 ± 0.44^{b}	> 0.1	77.56 ± 3.27^{b}	> 0.1	16.45 ± 0.74^{c}	< 0.1	10.67 ± 0.28^{c}	< 0.1
Ethyl acetate	85217.39 ± 1642.03^{c}	> 1	100109.49 ± 511.71^{a}	> 1	91657.95 ± 1951.33^{b}	> 1	28442.57 ± 386.25^{d}	> 1	25651.39 ± 187.95^{d}	> 1
Ethyl hexanoate	2809.75 ± 58.57^{a}	> 1	2316.84 ± 36.07^{b}	> 1	1902.3 ± 75.19^{c}	> 1	852.22 ± 20.31^{d}	> 1	723.14 ± 29.75^{d}	> 1
Ethyl lactate	22258.31 ± 199.5^{c}	> 0.1	24399.88 ± 226.65^{b}	> 0.1	25703.1 ± 222.29^{a}	> 0.1	11457.54 ± 91.99^{d}	> 0.1	9364.33 ± 3.71^{e}	> 0.1
Ethyl caprylate	3775.29 ± 26.11^{a}	> 1	2696.12 ± 29.21^{b}	> 1	2337.49 ± 99.08^{c}	> 1	1191.49 ± 27.25^{d}	> 1	926.69 ± 58.14^{e}	> 1
Ethyl nonanoate	4.46 ± 0.33^{a}	> 0.1	3.59 ± 0.34^{ab}	> 0.1	2.64 ± 0.35^{b}	> 0.1	2.41 ± 0.34^{b}	> 0.1	2.87 ± 0.74^{ab}	> 0.1
Ethyl butanoate	837.32 ± 25.93^{a}	> 1	746.62 ± 11.49^{b}	> 1	662.75 ± 21.04^{c}	> 1	174.02 ± 2.25^{d}	> 0.1	31.36 ± 1.42^{e}	> 0.1
Diethyl succinate	163.23 ± 1.66^{d}	< 0.1	200.21 ± 4.55^{c}	< 0.1	285.03 ± 0.25^{a}	< 0.1	201.89 ± 4.82^{c}	< 0.1	244.32 ± 4.13^{b}	< 0.1
Ethyl laurate	126.1 ± 7.08^{a}	> 0.1	65.46 ± 1.67^{b}	> 0.1	42.49 ± 1.97^{c}	< 0.1	13.2 ± 0.24^{d}	< 0.1	17.22 ± 2.07^{d}	< 0.1
Ethyl palmitate	95.75 ± 2.97^{a}	> 0.1	63.45 ± 1.18^{b}	< 0.1	59.5 ± 1.07^{bc}	< 0.1	40.86 ± 4.6^{c}	< 0.1	64.63 ± 12.03^{b}	< 0.1

Volatile compounds	100NTU	OAV	200NTU	OAV	300NTU	OAV	Muddy juice	OAV	Pulp	OAV
Ethyl 2-methylbutyrate	18.71 ± 0.8[ab]	> 0.1	16.92 ± 0.17[b]	> 0.1	20.47 ± 1.12[a]	> 0.1	10.25 ± 0.29[c]	> 0.1	8.36 ± 0.82[c]	> 0.1
Methyl octanoate	18.72 ± 0.25[a]	< 0.1	12.5 ± 0.03[c]	< 0.1	15.97 ± 0.67[b]	< 0.1	8.37 ± 0.23[c]	< 0.1	5.48 ± 0.38[d]	< 0.1
Isopentyl hexanoate	2.07 ± 0.08[a]	< 0.1	1.43 ± 0.02[b]	< 0.1	1.2 ± 0.18[b]	< 0.1	0.71 ± 0.03[c]	< 0.1	0.52 ± 0.04[c]	< 0.1
Isobutyric acid	1794.69 ± 156.66[c]	< 0.1	2005.01 ± 157.96[bc]	> 0.1	2655.05 ± 10.61[a]	> 0.1	2407.72 ± 136.37[ab]	> 0.1	2541.46 ± 7.58[a]	> 0.1
Hexanoic acid	5788.83 ± 66.87[a]	> 0.1	5118.28 ± 124.82[b]	> 0.1	4992.45 ± 170.87[b]	> 0.1	2692.75 ± 16.33[c]	> 0.1	2177.03 ± 40[d]	> 0.1
Octanoic acid	6615.25 ± 228.4[a]	> 0.1	5785.77 ± 166.61[b]	> 0.1	5866.71 ± 188.97[b]	> 0.1	3325 ± 34.4[c]	> 0.1	2259.82 ± 85.26[d]	> 0.1
Decanoic acid	932.77 ± 133.67[a]	< 0.1	676.94 ± 45.24[b]	< 0.1	617.91 ± 11.41[b]	< 0.1	555.35 ± 16.27[bc]	< 0.1	363.68 ± 33.54[c]	< 0.1
Decanal	5.92 ± 0.37[a]	> 1	4.48 ± 0.35[a]	> 1	3.63 ± 1.28[a]	> 1	4.2 ± 2.04[a]	> 1	2.86 ± 0.58[a]	> 1
Nonanal	3.09 ± 0.49[a]	> 1	4.15 ± 0.01[a]	> 1	3.91 ± 0.64[a]	> 1	4.27 ± 0.85[a]	> 1	3.41 ± 0.15[a]	> 1
Benzyl alcohol	164.12 ± 0.55[a]	< 0.1	137.2 ± 2.48[b]	< 0.1	130.88 ± 8.91[b]	< 0.1	60.46 ± 0.35[c]	< 0.1	5.02 ± 0.76[d]	< 0.1
Phenethyl acetate	1257 ± 31.44[c]	> 1	1789.75 ± 39.36[b]	> 1	2186.08 ± 74.34[a]	> 1	1055.91 ± 3.28[d]	> 1	492.92 ± 13.86[e]	> 1
α-Pinoresinol	0.59 ± 0.01[c]	-	0.99 ± 0.08[cb]	-	1.43 ± 0.08[b]	-	2.58 ± 0.3[a]	-	2.82 ± 0.05[a]	-
Linalool	5.5 ± 0.28[c]	> 0.1	6.06 ± 0.37[c]	> 0.1	7.81 ± 0.05[b]	> 0.1	9.47 ± 0.15[a]	> 0.1	7.39 ± 0.28[b]	> 0.1

Note: Values are mean ± standard deviation. Different lowercase letters in the same row indicate significant differences ($p < 0.05$, Duncan test). "-" indicates that the reference threshold was not found for this aroma component.

Supplementary table 7. Volatile components in kiwi wine fermented with different clarified juices of the "Yate" variety (μg/L)

Volatile compounds	100NTU	OAV	200NTU	OAV	300NTU	OAV	Muddy juice	OAV	Pulp	OAV
Isoamyl alcohol	152647.56 ± 24385.79[c]	> 1	181165.93 ± 615.09[bc]	> 1	197230.67 ± 9045.87[ab]	> 1	205153.34 ± 3611.86[ab]	> 1	228331.45 ± 15769.49[a]	> 1
Isobutanol	57718.05 ± 11429.42[d]	> 0.1	68501.22 ± 1906.59[c]	> 0.1	74575.51 ± 2191.3[c]	> 0.1	77571.18 ± 2723.97[b]	> 0.1	86335.13 ± 2612.86[a]	> 1
1-Heptanol	6713.52 ± 392.58[b]	> 1	6421.47 ± 81.66[b]	> 1	7451.55 ± 15.47[a]	> 1	3007.7 ± 23.51[c]	> 1	3235.51 ± 46.46[c]	> 1
1-Octanol	35.75 ± 3.65[c]	< 0.1	52.59 ± 0.4	< 0.1	54.17 ± 1.29	< 0.1	51.63 ± 0.32	< 0.1	66.82 ± 0.38	< 0.1
1-Pentanol	20637.2 ± 1948.76[a]	> 1	20972.58 ± 538.63[a]	> 1	21698.84 ± 728.78[a]	> 1	11894.29 ± 110.24[b]	> 1	13950.35 ± 308.25[b]	> 1
1-Decanol	7.16 ± 0.67[b]	< 0.1	7.89 ± 0.1[b]	< 0.1	7.71 ± 0.64[b]	< 0.1	5.21 ± 0.14[c]	< 0.1	10.21 ± 0.47[a]	< 0.1
3-Methyl-1-pentanol	71.03 ± 2.69[d]	< 0.1	86.08 ± 2.24[c]	< 0.1	94.34 ± 2.96[c]	< 0.1	129.87 ± 4.25[a]	> 0.1	118.45 ± 0.11[b]	> 0.1
(E)-3-hexen-1-ol	268.03 ± 13.03[a]	> 0.1	174.82 ± 1.04[c]	> 0.1	220.04 ± 2.21[b]	> 0.1	223.48 ± 6.8[b]	> 0.1	148.37 ± 3.68[d]	> 0.1
Isoamyl acetate	9755.92 ± 879.01[a]	> 1	10078.93 ± 224.49[a]	> 1	11136.25 ± 165.83[a]	> 1	3769.87 ± 57.26[b]	> 1	4393.27 ± 30.71[b]	> 1
Hexyl acetate	782.11 ± 74.5[a]	> 1	592.75 ± 14.32[b]	> 0.1	674.7 ± 16.3[ab]	> 0.1	140.41 ± 0.9[c]	> 0.1	160.42 ± 3.5[c]	> 0.1
Ethyl acetate	77790.57 ± 6283.8[a]	> 1	81228.97 ± 965.61[a]	> 1	84458.9 ± 119.24[a]	> 1	38119.74 ± 675.22[b]	> 1	43667.89 ± 493.69[b]	> 1
Ethyl hexanoate	1433.15 ± 135.11[a]	> 1	1451.67 ± 37.41[a]	> 1	1504.32 ± 50.7[a]	> 1	834.44 ± 9.19[b]	> 1	983.5 ± 21.93[b]	> 1
Ethyl lactate	9231.68 ± 640.79[c]	> 0.1	10312.67 ± 113.19[bc]	> 0.1	10614.27 ± 265.57[b]	> 0.1	13011.1 ± 356.38[a]	> 0.1	12298.89 ± 130.9[a]	> 0.1
Ethyl caprylate	2095.84 ± 230.02[a]	> 1	2234.63 ± 54.81[a]	> 1	2236.84 ± 10.36[a]	> 1	838.12 ± 0.84[b]	> 1	1143.02 ± 41.17[b]	> 1
Ethyl nonanoate	2.7 ± 0.35[ab]	> 0.2	2.85 ± 0.15[ab]	> 0.3	3.17 ± 0.18[a]	> 0.4	0.84 ± 0.14[c]	< 0.1	2.04 ± 0.47[b]	< 0.1
Ethyl butanoate	125.4 ± 9.05[b]	> 0.1	155.14 ± 4.19[a]	> 0.1	165.73 ± 2.43[a]	> 0.1	77.95 ± 0.34[c]	> 0.1	65.28 ± 1.65[c]	> 0.1
Diethyl succinate	94.88 ± 9.53[d]	< 0.1	121.04 ± 0.89[c]	< 0.1	137.22 ± 3.42[c]	< 0.1	182.27 ± 0.44[b]	< 0.1	203.25 ± 1.49[a]	< 0.1
Ethyl laurate	27.48 ± 3.32[a]	< 0.1	25.41 ± 1.29[a]	< 0.1	21.83 ± 0.9[a]	< 0.1	6.25 ± 0.29[b]	< 0.2	22.42 ± 1.9[a]	< 0.3

Volatile compounds	100NTU	OAV	200NTU	OAV	300NTU	OAV	Muddy juice	OAV	Pulp	OAV
Ethyl palmitate	63.2 ± 11.72[ab]	< 0.1	50.48 ± 1.91[b]	< 0.1	45.02 ± 0.06[b]	< 0.1	34.5 ± 3.98[b]	< 0.1	93.87 ± 18.49[ab]	< 0.1
Ethyl 2-methylbutyrate	5.97 ± 0.54[c]	< 0.1	7.99 ± 0.14[b]	> 0.1	7.49 ± 0.28[b]	> 0.1	8.15 ± 0.07[b]	> 0.1	10.59 ± 0.11[a]	> 0.1
Methyl octanoate	17.12 ± 1.93[a]	< 0.1	14.51 ± 0.41[a]	< 0.1	16.59 ± 0.27[a]	< 0.1	5.2 ± 0.06[b]	< 0.1	4.88 ± 0.11[b]	< 0.1
Isopentyl hexanoate	0.92 ± 0.05[a]	< 0.1	1.02 ± 0.02[a]	< 0.1	0.99 ± 0.02[a]	< 0.1	0.56 ± 0.01[b]	< 0.1	0.96 ± 0.05[a]	< 0.1
Isobutyric acid	2614.48 ± 112.47[c]	> 0.1	3060.01 ± 116.85[b]	> 0.1	2885.24 ± 42.95[bc]	> 0.1	2655.1 ± 38.39[c]	> 0.1	3580.14 ± 9.82[a]	> 0.1
Hexanoic acid	3805 ± 273.04[c]	> 0.1	3984.23 ± 66.08[c]	> 0.1	4493.43 ± 87.12[a]	> 0.1	1931.36 ± 14.65[a]	> 0.1	2217.94 ± 5.26[a]	> 0.1
Octanoic acid	4523.74 ± 393.09[b]	> 0.1	5739.19 ± 636.2[ab]	> 0.1	6055.13 ± 255.22[a]	> 0.1	1696.69 ± 36.69[c]	> 0.1	1631.35 ± 71.42[c]	> 0.1
Decanoic acid	505.69 ± 59.76[b]	< 0.1	658.21 ± 45.43[a]	< 0.1	683.65 ± 34.63[a]	< 0.1	159.7 ± 11.07[c]	< 0.1	193.07 ± 18.94[c]	< 0.1
Decanal	4.42 ± 0.13[ab]	> 1	5.19 ± 0.27[a]	> 1	3.17 ± 0.55[b]	> 1	4.54 ± 0.04[ab]	> 1	3.07 ± 0.91[a]	> 1
Nonanal	3.25 ± 0.16[a]	> 1	4.17 ± 0.3[a]	> 1	3.02 ± 1.1[a]	> 1	4.98 ± 0.59[a]	> 1	4.41 ± 1.53[a]	> 1
Phenethyl acetate	1864.22 ± 191.27[c]	> 1	2427.2 ± 10.2[b]	> 1	2774.18 ± 88.68[a]	> 1	823.6 ± 1.46[d]	> 1	810.15 ± 18.98[d]	> 1
Furfural	63.63 ± 5.74[b]	> 1	64.57 ± 0.82[ab]	> 1	74.99 ± 1.66[ab]	> 1	68.03 ± 5.08[ab]	> 1	81.23 ± 6.06[a]	> 1
α-Pinoresinol	1.28 ± 0.17[d]	-	1.72 ± 0.22[cd]	-	2.14 ± 0.13[bc]	-	2.9 ± 0.05[a]	-	2.55 ± 0.08[ab]	-
Linalool	5.86 ± 0.45[c]	> 0.1	7.52 ± 0.16[b]	> 0.1	8.57 ± 0.01[a]	> 0.1	8.85 ± 0.12[a]	> 0.1	7.02 ± 0.37[b]	> 0.1

Note: Values are mean ± standard deviation. Different lowercase letters in the same row indicate significant differences ($p < 0.05$, Duncan test). "-" indicates that the reference threshold was not found for this aroma component.
